# A comprehensive method for amplicon-based and metagenomic characterization of viruses, bacteria, and eukaryotes in freshwater samples

**DOI:** 10.1186/s40168-016-0166-1

**Published:** 2016-07-11

**Authors:** Miguel I. Uyaguari-Diaz, Michael Chan, Bonnie L. Chaban, Matthew A. Croxen, Jan F. Finke, Janet E. Hill, Michael A. Peabody, Thea Van Rossum, Curtis A. Suttle, Fiona S. L. Brinkman, Judith Isaac-Renton, Natalie A. Prystajecky, Patrick Tang

**Affiliations:** 1 British Columbia Public Health Laboratory, British Columbia Centre for Disease Control, Vancouver, BC V5Z 4R4 Canada; 2 South Kensington Campus, Imperial College London, Sir Ernst Chain Building, London, SW7 2AZ UK; 3 Department of Earth, Ocean and Atmospheric Sciences, University of British Columbia, Vancouver, BC V6T 1Z4 Canada; 4 Department of Microbiology and Immunology, University of British Columbia, Vancouver, BC V6T 1Z4 Canada; 5 Department of Botany, University of British Columbia, Vancouver, BC V6T 1Z4 Canada; 6 Department of Veterinary Microbiology, Western College of Veterinary Medicine, University of Saskatchewan, Saskatoon, SK S7N 5B4 Canada; 7 Department of Molecular Biology and Biochemistry, South Science Building, Simon Fraser University, Burnaby, BC V5A 1S6 Canada; 8 Integrated Microbial Biodiversity Program, Canadian Institute for Advanced Research, Toronto, ON M5G 1Z8 Canada; 9 Department of Pathology and Laboratory Medicine, Faculty of Medicine, University of British Columbia, Vancouver, BC V6T 1Z4 Canada; 10 Department of Pathology, Sidra Medical and Research Center, PO Box 26999, Doha, Qatar

**Keywords:** Microbiome, Watersheds, Amplicon sequencing, Metagenomes, Metagenomics, Microbial fractions

## Abstract

**Background:**

Studies of environmental microbiota typically target only specific groups of microorganisms, with most focusing on bacteria through taxonomic classification of 16S rRNA gene sequences. For a more holistic understanding of a microbiome, a strategy to characterize the viral, bacterial, and eukaryotic components is necessary.

**Results:**

We developed a method for metagenomic and amplicon-based analysis of freshwater samples involving the concentration and size-based separation of eukaryotic, bacterial, and viral fractions. Next-generation sequencing and culture-independent approaches were used to describe and quantify microbial communities in watersheds with different land use in British Columbia. Deep amplicon sequencing was used to investigate the distribution of certain viruses (*g23* and RdRp), bacteria (16S rRNA and *cpn*60), and eukaryotes (18S rRNA and ITS). Metagenomic sequencing was used to further characterize the gene content of the bacterial and viral fractions at both taxonomic and functional levels.

**Conclusion:**

This study provides a systematic approach to separate and characterize eukaryotic-, bacterial-, and viral-sized particles. Methodologies described in this research have been applied in temporal and spatial studies to study the impact of land use on watershed microbiomes in British Columbia.

**Electronic supplementary material:**

The online version of this article (doi:10.1186/s40168-016-0166-1) contains supplementary material, which is available to authorized users.

## Background

Water is the most basic and important natural resource on our planet. While water is a renewable resource, an expanding population and increased land use create stress on the aquatic environment and threats to water quality [1–3]. Although there are many users of water, including animals, agriculture, and industry, the current emphasis for water quality assessment is testing at the tap for the purpose of human consumption rather than at the source watershed. Laboratory tests for fecal pollution use traditional culture-based methods to detect bacteria such as *Escherichia coli* and total coliforms. Not only are these methods slow and inaccurate due to differences in enumeration strategies [4], but also they measure only a fraction of the microorganisms in the sample [5, 6], missing important perturbations in the microbiota.

Environmental or human disturbances can lead to perturbations in the watershed microbiome including changes in the endogenous microorganisms or the introduction of human or animal fecal microbiota. These changes in community structure in combination with environmental parameters may pinpoint to the source of disturbance in water quality. Thus, a better understanding of the entire watershed microbiome and sources of pollution in watersheds will be critical for assessing microbial community changes and associated threats to both ecosystem and human health. Previous work has demonstrated that (i) niche environments such as watersheds have unique microbial taxa signatures and (ii) microbial markers can be used to detect microbial pollution in water [7, 8]. Still, the microbiomes of freshwater ecosystems have not been as comprehensively studied as have other aquatic environments such as marine ecosystems [9–11].

Next-generation sequencing and culture-independent approaches enable the detection of these perturbations and the identification of biomarkers for pollution detection and source attribution. There are multiple studies that have been conducted using culture-independent approaches such as deep amplicon sequencing of the 16S rRNA gene and shotgun metagenomics to characterize bacterial communities and assess water quality and the overall ecology in freshwater ecosystems [8, 12–15]. While these studies have identified microbial signatures of water quality, they are based upon the analysis of a specific gene or microbial fraction (mainly bacteria) leaving other microbial fractions largely unexplored. For instance, plant viruses can be good markers for human fecal contamination [16, 17] and bacteriophages can be used for microbial source tracking [18], demonstrating that surveys of watershed microbiomes need to expand beyond the typical bacterial 16S rRNA or single fraction studies.

To date, there is only one study that has characterized the different major microbial domains within the same environmental sample (soil) [19]. The present study describes a series of methods developed to more comprehensively characterize freshwater microbial communities (eukaryotes, bacteria, and viruses) as a single unit. Water samples from three non-interconnected watersheds in southwestern British Columbia affected by different land use (agricultural, urban, and protected sites) were concentrated and fractionated by size using filtration then characterized using amplicon sequencing and metagenomics (sequencing all the genetic material in a sample). Sequence-based metagenomics aimed for bacterial and viral communities, while deep amplicon sequencing included 18S rRNA gene, internal transcribed spacer (ITS) for eukaryotes, and 16S rRNA and chaperonin-60 (*cpn*60) genes for bacteria. Due to the lack of a universal gene in viruses, amplicon sequencing was used to study only selected DNA and RNA viruses. Gene 23 (*g23*), which encodes the major capsid protein of T4-like bacteriophages, has been widely used for phylogenetic studies in different environments including aquatic environments [10, 20–23]. All known RNA viruses employ an RNA-dependent RNA polymerase (RdRp) for replication [24]. As the largest group of RNA viruses, *Picornavirales* have been reported to infect a wide diversity of eukaryotes in aquatic environments [11, 25–28]; the RdRp gene from this order was selected to complement viral RNA metagenomes in watersheds.

Additionally, traditional bacterial markers of low water quality such as total coliforms and *E. coli* were also included as part of this study. These series of approaches were piloted in order to validate the laboratory methods and define the baseline microbiota in three differently affected watersheds of southwestern British Columbia. Ultimately, these methods will be applied in larger longitudinal studies to study the impact of land use on watershed microbiomes and identify novel biomarkers of water quality.

## Methods

### Sample collection

Forty-liter samples were collected in sterile plastic carboys from three different watersheds in southwestern British Columbia, each representing a different land use type (protected, agricultural, and urban). Sampling within each site was conducted in two to three locations (upstream, downstream, and at the “polluted” site). Table 1 summarizes the description of sampling sites. Land use was the primary determinant of watershed selection. Watersheds were selected in collaboration with provincial agencies and scientists who have conducted research in these locations. A total of seven samples were collected within a 1.5-month period (March–April 2012). Samples were pre-filtered in situ using a 105-μm spectra/mesh polypropylene filter (SpectrumLabs, Rancho Dominguez, CA) and kept at 4 °C for transport to the laboratory for processing and storage within 2 h of the last sample collection. Ten liters of ultrapure (type 1) water (Milli-Q, Millipore Corporation, Billerica, MA) was used as a filtration control.

**Table 1:** Description of sampling sites

Watershed	Site name	Average depth (m) at cross section	Average width (m) at cross section	Elevation from the sea level (m)	Water flow (m^3^/s)	Description
Urban^a, b^	UPL	0.17	1.26	119	0.06	At site of urban “pollution,” in residential area.
	UDS	0.14	2.68	8	0.29	Downstream of urban “pollution,” 1 km from UPL.
Agricultural^c^	AUP	0.16	1.71	118	0.16	Upstream of agricultural “pollution.” Not affected by agricultural activity, with minimal housing nearby.
	APL	0.79	7.33	10	2.11	At site of agricultural “pollution.” AUP is upstream of APL, separated by 9 km. Multiple farms near this site.
	ADS	1.72	25.5	8	9.97	Downstream of agricultural “pollution.” ADS is downstream of APL, 2.5 km away.
Protected	PUP	0.24	7.7	198	0.60	Upstream of drinking water reservoir in protected watershed.
	PDS	2.1	2.1	111	1.01	Downstream of PUP-fed reservoir, collected after passing through an 8.8 km pipe.

^a^Average distance between urban and agricultural watershed: 63 km

^b^Average distance between urban and protected watershed: 101 km

^c^Average distance between agricultural and protected watershed: 132 km

### Metadata

Physico-chemical water quality parameters were measured in situ using a YSI Professional Plus handheld multiparameter instrument (YSI Inc., Yellow Springs, OH), a VWR turbidity meter model No. 66120-200 (VWR, Radnor, PA) and a Swoffer 3000 current meter (Swoffer Instrumentsz, Seattle, WA). Total coliform and *E. coli* counts were determined using Colilert-24 (IDEXX Laboratories, Westbrook, ME). Chemical analysis included dissolved chloride (mg/L) and ammonia (mg/L) using automated colorimetric (SM-4500-Cl G) and phenate methods (SM-4500-NH3 G) [29]. Additionally, nutrients (orthophosphates, nitrites, and nitrates) were analyzed following methods described by Murphy and Riley [30] and Wood et al. [31], respectively.

### Fraction separation

Microbial fractions were separated through a combination of serial filtration approaches. Following pre-filtration in situ, water was filtered through a 1-μm Envirochek HV (Pall Corporation, Ann Harbor, MI) sampling capsule to capture eukaryotic-sized particles, followed by filtration through a 0.2-μm 142-mm Supor-200 membrane disc filters (Pall Corporation, Ann Harbor, MI) to capture the bacterial-sized particles. To remove any remaining bacterial cells, the permeate was filtered again using a 0.2-μm Supor Acropak 200 sterile cartridge (Pall Corporation, Ann Harbor, MI) prior to tangential flow filtration (TFF). Viral-sized particles were concentrated to approximately 450 mL as described by Suttle et al. [32] and Culley et al. [26], using a regenerated cellulose Prep/Scale TFF cartridge (Millipore Corporation, Billerica, MA) with a 30-kDa molecular-weight cutoff and nominal filter area of 0.23 m^2^.

### Collection, fixation, and particle quantitation of environmental samples using flow cytometry (FCM)

Nine hundred and eighty-microliter aliquots of raw water and 0.2 μm permeate, ultrafiltrate, and viral concentrate were collected in duplicates during the filtration process. Samples were fixed with 20 μl of 25 % glutaraldehyde to reach a final concentration of 0.5 % glutaraldehyde, inverted to mix, incubated at 4 °C in the dark for 20 min, and then transferred to −80 °C freezer for storage and further analysis. Abundance of viral and bacterial-sized particles were determined in duplicate water samples using a FACSCalibur flow cytometer (Beckton Dickinson, San Jose, CA) with a 15-mW 488-nm air-cooled argon-ion laser as described by Brussaard (2004) [33]. Analysis of the FCM results was conducted using CYTOWIN version 4.31 (2004) [34].

### Elution and concentration of microbial cells and viral particles

Mechanical procedures involving shaking and centrifugation were used to remove and concentrate microbial cells from the filters. Cells were washed with ×1 phosphate-buffered solution (PBS) and 0.01 % Tween pH 7.4. Eukaryotic cells retained in the 1-μm Envirochek HV capsules were eluted according to the manufacturer’s protocol (Pall Corporation, Ann Harbor, MI). Eluates (~500 μL) of eukaryotic cells were dispensed into 1.7-mL microcentrifuge tubes and further precipitated by centrifugation (15 min, 1500×*g*, 4 °C). Samples were kept at −80 °C for further nucleic acid extraction.

To minimize the number of DNA extraction tubes, the 0.2-μm Supor membrane disc filter(s) was washed with 15 mL of PBS to remove bacterial cells followed by centrifugation (15 min, 3300×*g*, 4 °C). Aliquots of the washed cell suspension were stored at −80 °C for further DNA extraction. Viral-sized particles eluted in 450 mL of sample required further concentration by ultracentrifugation (4 h, 121,000×*g*, 4 °C). Viral-sized concentrate pellets were resuspended in ×1 PBS to reach a final volume of approximately 5 to 6 mL and incubated overnight at 4 °C with constant agitation (180 rpm). An evaluation of ultracentrifugation as an approach to further concentrate viral-sized particles is also described here.

### Concentration of viral particles by ultracentrifugation

Validation of ultracentrifugation as a method to isolate virus-like particles was conducted using two DNA and RNA viruses isolated from clinical specimens at the British Columbia Centre for Disease Control (BCCDC): adenovirus (90–100 nm) and enterovirus (Coxsackie B2, ~30 nm). Both viruses are routinely used as controls at the BCCDC. An aliquot of 0.25 μl of adenovirus and enterovirus control stocks was inoculated into A549 and primary *Rhesus* monkey kidney cell cultures (Diagnostic Hybrids, Athens, OH), respectively. Once the cytophatic effect was 3+, cells were harvested in minimal essential media (MEM) with 2 % fetal calf serum (Sigma-Aldrich, St. Louis, MO), separately brought up to a final volume of 16 mL, and stored at −80 °C until later processing. For further cell lysis and release of viral particles, samples were subjected to three rounds of freeze-thaw. Following the final thaw, samples were filtrated through a 0.2-μm Supor membrane syringe filters (Pall Corporation, Ann Harbor, MI) and spiked with 435 mL of MEM. The recovery efficiency was evaluated for both supernatant and concentrated pellets at different time points (1, 2, and 4 h) of the ultracentrifugation process (121,000×*g*, 4 °C). Virus concentrate pellets were incubated overnight at 4 °C on a shaker. At least duplicate aliquots from the different stages of the previously described processes were collected for flow cytometry counts, nucleic acid extraction, and quantitation of viruses in samples.

### Nucleic acid extraction of adenoviruses and enteroviruses

Samples collected throughout the ultracentrifugation process were pre-treated with 1× RNAsecure (Life Technologies, Carlsbad, CA) and 5 units (U) of DNase I (Epicentre Biotechnologies, Madison, WI). This reaction was terminated by adding 10 mM EDTA (pH 8.0) for 15 min at 65 °C. DNA and RNA from adenoviruses and enteroviruses, respectively, from were extracted using the NucliSens easyMAG system (bioMérieux, Craponne, France). Nucleic acids were further precipitated using 0.1 volumes of 3-M sodium acetate and two volumes of 100 % ethanol, washed with 1 mL of ice-cold 70 % ethanol, and resuspended in 10 mM Tris solution. Nucleic acid concentration and purity was assessed with Qubit dsDNA high sensitivity and RNA assay kits in a Qubit 2.0 fluorometer (Life Technologies, Carlsbad, CA) and NanoDrop spectrophotometer (NanoDrop technologies, Inc., Wilmington, DE), respectively.

### Quantitative polymerase chain reaction (qPCR) of adenoviruses and enteroviruses

#### Quantitation of adenoviruses

Detection of adenoviruses was carried out using a combination of primers described by Wong et al., 2008 [35] (Table S1). These primer sets amplify a conserved region (81–87 bp) of the hAdV hexon gene. DNA extracted from raw samples was used as template to generate amplicons for standard curve. PCR conditions were conducted as follows: 94 °C for 5 min, followed by 35 cycles of 94 °C for 30 s, 53 °C for 30s, 72 °C for 30 min, and a final extension at 72 °C for 10 min. PCR amplicons were purified with a QIAQuick PCR Purification Kit (Qiagen Sciences, Maryland, MD) according to the manufacturer’s instructions.

#### Quantitation of enteroviruses

RNA (4 μl) extracted from raw samples was first treated with Turbo DNase I (Life Technologies, Carlsbad, CA) following the manufacturer’s instructions. RNA was then converted into complementary DNA (cDNA) using Superscript III reverse transcriptase (Life Technologies, Carlsbad, CA). Amplification of the UTRe gene in enteroviruses was conducted using primers described by Verstrepen et al. [36] and Watzinger et al. [37] (Table S1). This primer set amplifies a specific 148-bp region within this gene. cDNA from raw samples was used as template to generate amplicons for standard curve. PCR conditions were conducted as follows: 94 °C for 5 min, followed by 35 cycles of 94 °C for 30 s, 51 °C for 30s, 72 °C for 30 min, and a final extension at 72 °C for 10 min. PCR amplicons were purified with a QIAQuick PCR Purification Kit (Qiagen Sciences, Maryland, MD) according to the manufacturer’s instructions.

Standard curves for adenoviruses and enteroviruses were generated by ligating purified amplicons of adenoviruses and enteroviruses into pCR2.1-TOPO cloning vectors (Invitrogen) and transformed into One Shot *E. coli* DH5α-T1R competent cells following the manufacturer’s protocol. One transformant was selected and grown overnight at 37 °C in LB broth with 50 μg/mL of kanamycin. Plasmids were extracted and purified using Purelink Quick Plasmid Miniprep kit (Life Technologies, Carlsbad, CA) and quantified using Qubit dsDNA high sensitivity assay kit (Life Technologies, Carlsbad, CA). Plasmid DNA was linearized by digestion with the BamHI-HF endonuclease (New England BioLabs Inc., Ipswich, MA). Serial dilutions of the linearized plasmid were used as templates to generate standard curves for qPCR and RT-qPCR. Each 20-μl real-time PCR mixture consisted of 10 μl of Fast SYBR Green Master Mix (2X) Real-Time PCR Master Mix (Life Technologies, Carlsbad, CA), 250 nM each primer, and 1 μl of template DNA or cDNA. The thermal cycling conditions consisted of initial denaturation for 20 s at 95 °C, followed by 40 cycles of 3 s at 95 °C and 20 s at 60 °C. Gene copy numbers for each sample were run in triplicate using a 7900 HT Fast Real-Time PCR system (Life Technologies, Carlsbad, CA). To verify the absence of non-specific amplification, a dissociation step was included and amplicons were analyzed on a 1.5 % agarose gel.

### Nucleic acid extraction and quality controls

Before extraction and to facilitate disruption of eukaryotic cells, eight freeze-thaw cycles, followed by overnight proteinase K digestion (Qiagen Sciences, Germantown, MD), were conducted for this fraction [38]. DNA was extracted from eukaryotes and bacterial cell fractions using the UltraClean Soil DNA Kit (MoBio, Carlsbad, CA) as per the manufacturer’s instructions. Concentrated viral-sized particles were pre-treated with 1X RNAsecure (Life Technologies, Carlsbad, CA) and 5 U of DNase I (Epicentre Biotechnologies, Madison, WI). This reaction was terminated with 10 mM EDTA (pH 8.0) for 15 min at 65 °C. Total nucleic acids were extracted from the viral fraction using the NucliSens easyMAG system (bioMérieux, Craponne, France). Nucleic acids from all fractions were further precipitated using 0.1 volumes of 3-M sodium acetate, two volumes of 100 % ethanol, and 5 μl of 5 μg/μl linear acrylamide. Samples were stored at −80 °C overnight then centrifuged at 17,000×*g* for 30 min at 4 °C. Supernatants were discarded, and pellets were washed with 70 % ice-cold ethanol, air dried, and resuspended in 10 mM Tris Cl, pH 8.5. Concentration, purity, and average size of nucleic acids were assessed with Qubit dsDNA High Sensitivity or RNA Assay kits in a Qubit 2.0 fluorometer (Life Technologies, Carlsbad, CA), NanoDrop spectrophotometer (NanoDrop Technologies, Inc., Wilmington, DE), and Agilent High Sensitivity DNA kit (Agilent Technologies, Inc., Santa Clara, CA), respectively.

Cysts and oocysts from *Giardia lamblia* and *Cryptosporidium parvum* (Waterborne, Inc., New Orleans, LA), respectively, were used as positive control for DNA extraction and amplification of the 18S rRNA gene. An isolate of *Aspergillus flavus* was used as a control for amplification of the ITS region. A strain of *E. coli* (ATCC 25922) was used as positive control for 16S rRNA and *cpn*60 genes. For DNA viruses and *g23* gene, a myovirus propagated in *Synechococcus sp*. strain WH7803 was used as a positive control. As a positive control for RNA viruses and RdRp amplicons, cultures of *Heterosigma akashiwo* were grown and infected with HaRNAV (isolate SOG263). Negative controls included sterile water and PBS.

### cDNA synthesis and random amplification of the viral fraction

A modified adapter nonamer approach described by Wang et al. [39] was used for cDNA synthesis and increase yields of the viral fraction. An aliquot of 4 μl from the total nucleic acids in the viral fraction was treated with Turbo DNase I (Life Technologies, Carlsbad, CA), following the manufacturer’s instructions. DNAsed samples (RNA) were then converted to cDNA using random nonamer primer A (5′-GTTTCCCACTGGAGGATA-N9-3′) and Superscript III reverse transcriptase (Life Technologies, Carlsbad, CA). Second strand synthesis was carried out using two rounds of Sequenase Version 2.0 DNA Polymerase (Affymetrix, Santa Clara, CA). Samples were stored at −20 °C until further processing. Subsequently, 5 μl of double-stranded cDNA samples was used as templates in a 50-μl PCR reaction consisting of 5 U of KlenTaq LA polymerase, 1X Klentaq PCR buffer, 0.2-mM nucleotides, 2 μM of primer B (5′-GTTTCCCACTGGAGGATA-3′). Random amplification was carried out as follows: 94 °C for 4 min, 68 °C for 5 min followed by 30 cycles of 94 °C for 30 s, 50 °C for 1 min, and 68 °C for 1 min and a final extension at 68 °C for 2 min. The amplified material was then cleaned up with Agencourt AMPure XP-PCR purification system (Beckman Coulter Inc., Brea, CA) at a 1.8× ratio. Primer B was excised using 4 U of BpmI (New England BioLabs Inc., Ipswich, MA). Digested products were cleaned up with Agencourt AMPure XP-PCR purification system (Beckman Coulter Inc., Brea, CA) at a 1.8× ratio. Finally, samples were end-repaired using 0.2-mM nucleotides, 1× T4 ligase buffer, 3 U of T4 DNA polymerase, 5 U of DNA polymerase I large (Klenow) fragment, and 10 U of T4 polynucleotide kinase (New England BioLabs Inc., Ipswich, MA). For random amplification of viral DNA, the random nonamer primer A and Sequenase DNA Polymerase were used as described above. Fragments generated in the random amplification process were further analyzed using the Agilent High Sensitivity DNA Kit (Agilent Technologies, Inc., Santa Clara, CA) and quantified using the Qubit dsDNA High Sensitivity Assay Kit (Life Technologies, Carlsbad, CA).

### Amplification of gene targets

Table 2 summarizes the primer sets and conditions used for the generation of amplicons described in the present study. Nucleic acids extracted from water samples and controls were analyzed for V1–V3 regions of the 18S rRNA gene and internal transcribed spacer (ITS1/ITS2) region for eukaryotes; hypervariable V3–V4 regions of the 16S rRNA and *cpn*60 genes for bacteria; and *g23* for T4-like bacteriophages and the RdRp gene for picorna-like viruses. Each PCR reaction consisted of 1.5-mM MgCl_2_, 0.2-mM nucleotides, 0.4 μM of primers, 1.25 U of Hot Start Polymerase (Promega Corporation, Fitchburg, WI), 1:10 dilution of template DNA, and water in a 50-μl volume. Fragments of the *cpn*60 gene were amplified using a primer mixture containing a 1:3 M ratio of primers H279/H280 and primers H1612/H1613 as described by Schellenberg et al. [46]. RNA-dependent RNA polymerase genes were amplified using Illustra Ready-To-Go PCR Beads (GE Healthcare UK Limited, Buckinghamshire, UK), 0.4 μM of primers, 1 μl of randomly amplified viral cDNA, and water in a 25-μl volume. PCR amplicons were run in duplicates, examined in a 1.5 % agarose/0.5X TBE gel stained with 1X GelRed (Biotium, Inc., Hayward, CA), and purified with a QIAQuick PCR Purification Kit (Qiagen Sciences, Maryland, MD) according to the manufacturer’s instructions.

**Table 2:** Description of primers used in PCR and quantitative PCR

Target gene	Primer name and sequences (5′ ➔ 3′)	Amplicon size (bp)	Thermal program		References
18S rRNA	EuK1A: CTGGTTGATCCTGCCAG 499R: CACCAGACTTGCCCTCYAAT	~500	94 °C × 5 min, 35 cycles of 30 s at 94 °C, 60 s at 55 °C, and 90 s at 72 °C, and a final cycle of 10 min at 72 °C.		[40, 41]
ITS	ITS1: TCCGTAGGTGAACCTGCGG ITS4: TCCTCCGCTTATTGATATGC	~500	95 °C × 15 min, 35 cycles of 30 s at 95 °C, 30 s at 55 °C, and 90 s at 72 °C, and a final cycle of 10 min at 72 °C.		[42]
β-tubulin (qPCR)	BT107F: AACAACTGGGCIAAGGTYACTACAC BT261R: ATGAAGAAGTGGAGICGIGGGAA	~450	Initial denaturation 20 s at 95 °C, followed by 40 cycles of 1 s at 95 °C and 30 s at 60 °C.		[43]
16S rRNA	341F: CCTACGGGAGGCAGCAG R806: GGACTACHVGGGTWTCTAAT	~465	94 °C × 5 min, 35 cycles of 45 s at 94 °C, 45 s at 50 °C, and 60 s at 72 °C, and a final cycle of 10 min at 72 °C.		[44, 45]
*cpn60*	H279: GAIIIIGCIGGIGAYGGIACIACIAC H280: YKIYKITCICCRAAICCIGGIGCYTT H1612: GAIIIIGCIGGYGACGGYACSACSAC H1613: CGRCGRTCRCCGAAGCCSGGIGCCTT	~578	3 min at 94 °C, 40 cycles of 30 s at 94 °C, followed by a temperature gradient of 1 min at 42 °C, 48 °C, 54 °C, or 60 °C, and 1 min at 72 °C, followed by a final extension of 10 min at 72 °C.		[46]
16S rRNA (qPCR)	341F: CCTACGGGAGGCAGCAG 518R: ATTACCGCGGCTGCTGG	~194	Incubation 2 min at 50 °C. Initial denaturation 20 s at 95 °C, followed by 40 cycles of 1 s at 95 °C and 20 s at 60 °C.		[44]
*uidA* (qPCR)	784F: GTGTGATATCTACCCGCTTCGC 866R: GAGAACGGTTTGTGGTTAATCAGGA EC807: FAM-TCGGCATCCGGTCAGTGGCAGT-BHQ1	84	Incubation 2 min at 50 °C. Initial denaturation 10 min at 95 °C, followed by 40 cycles of 15 s at 95 °C and 1 min at 60 °C.		[47]
*g23* (qPCR)	MZIA1bis: GATATTTGIGGIGTTCAGCCIATGA MZIA6: CGCGGTTGATTTCCAGCATGATTTC	~471	94 °C × 1.5 min, 35 cycles of 45 s at 94 °C, 60 s at 50 °C, and 60 s at 72 °C, and a final cycle of 5 min at 72 °C.	Incubation 2 min at 50 °C. Initial denaturation for 20 s at 95 °C, 40 cycles of 1 s at 95 °C and 30 s at 60 °C.	[20]
RdRp	RdRp1: GGRGAYTACASCIRWTTTGAT RdRp2: MACCCAACKMCKCTTSARRAA	~450	94 °C × 75 s, 40 cycles of 45 s at 94 °C, 45 s at 50 °C, and 60 s at 72 °C, and a final cycle of 5 min at 72 °C.		[26]

### Quantitative polymerase chain reaction of eukaryotes, bacteria, *E. coli*, and T4-type bacteriophages

Estimates of eukaryotes, bacteria, *E. coli*, and T4-type bacteriophage quantities in watershed sites were determined using β-tubulin, 16S rRNA, *uidA*, and *g23* gene fragments, respectively (Table 2). Gene copy numbers were calculated as previously described by Ritalahti et al. [48]. A modification based on sample dilution and volume was introduced to this calculation in terms of GCNs per milliliter sample. Standard curves for qPCR were generated using serial dilutions of linearized pCR2.1-TOPO vector (Life Technologies, Carlsbad, CA) with either cloned β-tubulin, 16S rRNA, and *g23* genes. *E. coli* genomic DNA was used for standard curves for *uidA* gene. Each 20-μl real-time PCR mixture consisted of 10 μl of Fast SYBR Green Master Mix (2X) Real-Time PCR Master Mix, 250 nM of each primer, and 1 μl of template DNA. Quantitation of the *uidA* gene fragment used Taqman Universal PCR Master Mix (Life Technologies, Carlsbad, CA) and followed the conditions, oligonucleotides (400 nM), and probe (200 nM) concentrations described by Maheux et al [49]. SYBR green-labeled reactions were conducted on a 7900 HT Fast Real-Time PCR system (Life Technologies, Carlsbad, CA), while Taqman-labeled reactions were carried out on a 7500 Fast Real-Time PCR system (Life Technologies, Carlsbad, CA). Each qPCR was run in triplicate. To verify the absence of non-specific amplification, a dissociation step was included in the SYBR green-labeled reactions, and amplicons were visualized on a 1.5 % agarose gel.

### DNA library preparation and sequencing

Libraries of 18S rRNA, ITS, 16S rRNA, *g23*, and RdRp amplicons were prepared using the NEXTflex ChIP-Seq Kit (BIOO Scientific, Austin, TX) with the gel-size selection option provided in the manufacturer’s instructions. The universal target region of the *cpn*60 gene was amplified using a 1:3 primer cocktail of H279/H280:H1612/H1613 as previously described by Schellenberg et al. [46].

Bacterial genomic DNA libraries were prepared using the Nextera XT DNA sample preparation kit (Illumina, Inc., San Diego, CA). One nanogram of bacterial DNA was fragmented following the manufacturer’s instructions. Libraries from randomly amplified viral DNA and cDNA fractions were prepared using NEXTflex ChIP-Seq kit (BIOO Scientific, Austin, TX) by following a gel-free option provided in the manufacturer’s instructions.

Amplicon, bacterial, and viral library sequencing were performed on an Illumina MiSeq (Illumina, Inc., San Diego, CA) using MiSeq reagent kits V2 with 150- and 250-bp paired-end outputs. *cpn*60 pyrosequencing libraries were sequenced on a Roche 454 Genome Sequencer FLX Titanium following standard protocols (Laboratory for Advanced Genome Analysis, Vancouver Prostate Centre). Additionally, PhiX sensu lato, an adapter-ligated ssDNA virus was used as control in Illumina sequencing. Amplicon libraries used 5 % PhiX, while that for bacterial and viral metagenome libraries used 1 % PhiX.

Amplicon and metagenomic sequencing control genomic DNA from four bacterial strains was used as 16S rRNA gene amplicon and metagenomic sequencing control. Bacterial mock community included Nocardioides *sp*. JS614, *Pseudomonas aeruginosa* PA01, *Rhodobacter capsulatus* SB1003, and *Streptomyces coelicolor* A3. Viral mock community consisting of genomic DNA and cDNA from myovirus and HaRNAV as well as *g23* and RdRp amplicons was used as sequencing controls. Bacterial and viral mock communities were pooled in equal molar concentrations, indexed, and sequenced with the environmental samples described in this study. Sequencing controls were not included for the eukaryotic fraction (18S rRNA and ITS).

### Data analysis

Gene copy number (GCN) or flow cytometry count (FCM) data were log_10_ transformed for analysis. One-way analysis of variance was run using Statistical Analysis System (SAS, version 9.1.3 for Windows) on the qPCR and FCM data to detect differences among target microbial fractions. Tukey’s test was used to determine statistical differences among the different sites. Correlations were assessed using Spearman correlation coefficients. A *p* value of 0.05 was assumed for the test as a minimum level of significance.

Adapter and primer sequences of amplicon and viral libraries were removed using Cutadapt [50], while short (<100 bp)- and low-quality reads were discarded using Trimmomatic version 0.32 [51]. Forward reads of amplicon and viral libraries were uploaded to the Metagenomic Rapid Annotations using Subsystems Technology (MG-RAST) [52] and Metavir [53], respectively. Bacterial amplicon analysis was also performed using QIIME [54] to identify trends robust to analysis platform. The raw data from *cpn*60 amplicon sequencing was processed through microbial profiling using metagenomic assembly (mPUMA) pipeline [55]. Bacterial metagenome sequence reads were trimmed using Adapter and AdapterRead2 parameters embedded in the MiSeq Reporter software (Illumina, Inc., San Diego, CA). Furthermore, paired-end sequences were merged using PANDAseq [56] and then uploaded to the MG-RAST pipeline [53]. Short (<151 bp) and unmerged bacterial metagenomic reads were discarded.

Taxonomic classifications for eukaryotic and bacterial amplicon and bacteria metagenomic sequence reads were based on the lowest common ancestor method [57]. The MG-RAST bacterial metagenomic results were subsequently confirmed by analysis with MEGAN4 [58]. For viral reads, taxonomic composition was computed using BLASTx from the NCBI website and adjusted via length normalization using the Genome relative Abundance and Average Size (GAAS) Metagenomic Tool [59]. Functional gene composition for bacterial and viral metagenomes was annotated using MG-RAST and the SEED subsystems [60]. A minimum percent identity of 60 % and annotations with an *e* value cutoff of 10^-3^ or less were used for further analyses. Microbial diversity and richness indexes were calculated using EstimateS (version 9.1.0) [61], available from http://viceroy.eeb.uconn.edu/estimates/. Multivariate analysis was performed for bacterial and viral metagenomes and amplicons using the Bray-Curtis metric.

## Results and discussion

Approximately 40 L of raw water was collected from watershed sites in BC during a 1.5-month period (Spring 2012). A combination of conventional and tangential flow filtration was used to separate eukaryotic-, bacterial-, and viral-sized particles, followed by nucleic acid extraction for these microbial fractions. The utility of the protocol was tested in terms of the quality of the resulting sequence libraries and the ability to characterize the microbial communities. Additional file 1: Table S2 summarizes the water quality parameters measured at each watershed location.

### Efficiency of filtration of microbial communities

Dead end and tangential flow filtration (TFF) have widely been used for the separation of microbial communities in water [26, 32, 62]. A significant correlation (96.1 %, *p* ≤ 0.0007) was observed between viral-like particles and bacterial cell counts by flow cytometry (Additional file 1: Table S3). Flow cytometry counts in raw water detected between 5.03 × 10^6^ and 1.18 × 10^8^ virus-like particles per milliliter of sample, while bacterial counts ranged between 1.55 × 10^5^ and 1.24 × 10^6^ cells/mL of environmental water. Virus-like particles were significantly higher (*p* < 0.0001) in APL compared to other watershed locations. Bacterial cell counts in APL were higher compared to watershed locations (*p* < 0.0001), except ADS (*p* = 0.4231). Overall, TFF was able to achieve a 94-fold concentration of the viral fraction from an initial volume of ~38.7 L to an average final volume of 415 mL. Viral concentration efficiency averaged 6 ± 51 % while bacterial concentration efficiency averaged 90 ± 11 %. The wide range in viral recovery efficiencies may be associated with losses during filtration [63–65]. Water with high turbidity and suspended solids tend to saturate filters [66, 67], and lower recovery efficiencies were observed in agricultural samples (APL and ADS), where turbidity and total dissolved solids values were higher (Additional file 1: Table S2).

### Ultracentrifugation as a method to improve recovery of viruses

Assessment of ultracentrifugation to further concentrate viral particles was performed using qPCR and FCM for adenoviruses and enteroviruses spiked in different volumes of MEM. Comparable recovery efficiencies have been reported with ultracentrifugation [68, 69]. Additional file 1: Figure S1 depicts quantitation of adenovirus (A) and enterovirus (B) per milliliter sample throughout different stages of the ultracentrifugation process and over time (1, 2, and 4 h). A gradual decrease in terms of viral GCNs and particles per milliliter was observed in supernatants collected at different time points of 1, 2, and 4 h using both approaches.

Recovery efficiency as measured by qPCR was estimated to be 54.2 and 68.2 % for adenoviruses and enteroviruses, respectively. Recovery efficiencies were also determined by flow cytometry with average percentages of 160 ± 26.3 % for adenoviruses and 0.5 ± 0.1 % for enteroviruses. Correlation analysis between qPCR approach and flow cytometry counts detected coefficients of 0.9206 (*p* = 0.0004) and 0.8683 (*p* = 0.0024) for adenoviruses and enteroviruses, respectively (Additional file 1: Figure S2). The observed differences between qPCR and FCM to quantify virus particles for enteroviruses may be associated to FCM underestimating ssRNA viruses <30 nm in diameter [70, 71]. In this work, we used Coxsackie B2 enterovirus, which is approximately 30 nm. This may indicate that only a fraction of this enterovirus was measurable by FCM as compared to the qPCR approach. It is also possible that some of these cells containing viruses may have been caught in the 0.2-μm filters. This extra step was conducted to filter out cell debris as well as simulate the filtration system used in this research. Although qPCR approach seemed to be more sensitive to detect adenoviruses and enteroviruses in this validation experiment, the lack of a highly conserved viral gene makes the quantitation of viruses difficult compared to other microbial fractions such as bacteria or eukaryotes. Thus, FCM was the method chosen to monitor viral-like particles in water samples. In this study, recovery rates using ultracentrifugation to concentrate virus-like particles in watershed samples and quantified using FCM were between 52.9 and 114.8 % (urban sites, data not shown).

### Nucleic acid yields and quality assessment

Although the nucleic acid yields from this study were compared to other similar studies, direct comparisons are difficult given the differences in water matrix conditions and procedures used (Table S4). Overall nucleic acid yields (excluding viral RNA fraction) had the same order of magnitude across the different filter pore sizes used in this study (Table S4). Total RNA extracted from the viral-sized fraction could only be detected in agricultural sites.

Nucleic acid purity was also estimated (Table S4). The A_260_/A_280_ and A_260_/A_230_ ratios (that indicate potential protein and humic acid contamination) were >1.4 and between 0.5 and 2.1, respectively. Similar results have been reported for A_260_/A_280_ and A_260_/A_230_ ratios using commercial kits and automated platforms for nucleic acid extraction from environmental samples [72–76]. While the A_260_/A_230_ ratio suggested humic acid contamination, it did not inhibit downstream applications such as PCR, qPCR, random amplification, library preparation, and sequencing.

### Amplification and quantitation of microbial fractions

#### Polymerase chain reaction

The utility of the protocol was tested using a PCR-based targeted sequencing approach for all three fractions. While 18S rRNA and ITS (eukaryotes), 16S rRNA and *cpn*60 (bacteria), and *g23* (T4-type bacteriophage) were detected in all watershed sites, RdRp amplicons (picorna-like viruses) could only be detected in agricultural sites (AUP, APL, and ADS) and the urban downstream site (UDS). Picorna-like viruses have been reported in British Columbia waters and mainly coastal waters infecting eukaryotic phytoplankton [28, 77]. In this study, RdRp fragments were found in watershed sites where dissolved solids and turbidity values were higher compared to other sites (Additional file 1: Table S2). Moreover, in experimental observations, RdRp fragments have been detected consistently over time in agricultural sites where conductivity and derived parameters such as salinity, specific conductance, and total dissolved solids are relatively higher (data not shown). The detection of these picorna-like viruses in a freshwater environment may also be attributable to terrestrial runoff or excretion by birds and fish [11, 78].

#### Random amplification

Viral RNA yields were lower compared to the viral DNA, eukaryotic, and bacterial fractions (Table 2). Although viruses are the most abundant entities in the environment, viruses only make up ~5 % of the relative biomass within microbial communities [79]. The small quantities of viral nucleic acids represent a challenge for downstream applications. Large volumes (from tens to hundreds of liters) of water are typically required to isolate and concentrate viral nucleic acids [11, 27, 80, 81]. The average fragment lengths of the amplified viral cDNA and DNA ranged from 200 to 2 kb with an average length of 400 bp (data not shown), which is similar to other viral studies [82, 83].

### Quantitation of microbial fractions

**Fig. 1:**
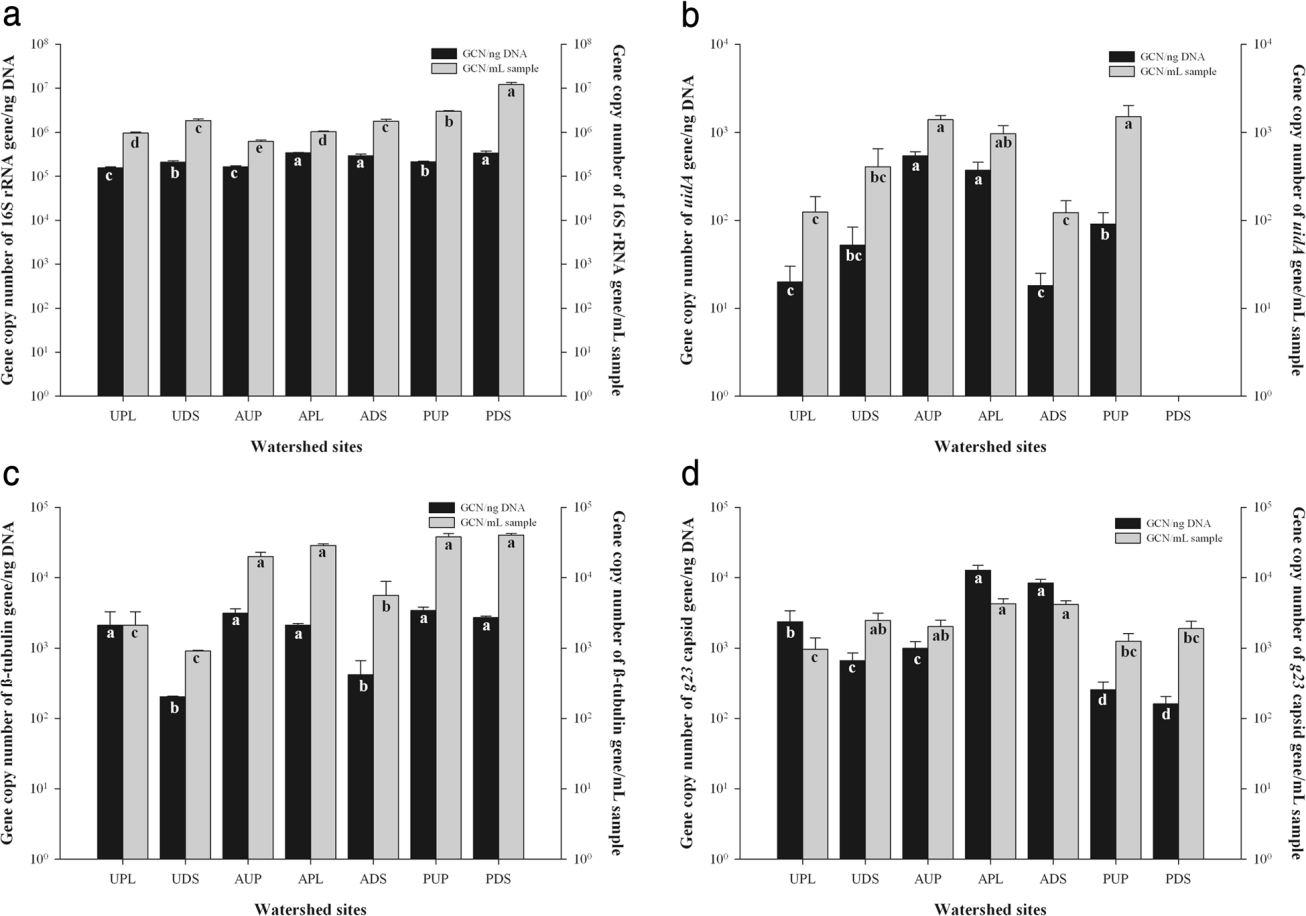
Gene copy numbers of 16S rRNA (**a**), *uidA* (**b**), β-tubulin (**c**), and *g23* (**d**) gene fragments detected in watershed sites. *UPL* urban polluted, *UDS* urban downstream, *AUP* agricultural upstream site, *APL* agricultural polluted, *ADS* agricultural downstream, *PUP* protected upstream, *PDS* protected downstream. *Black bars* represent the mean GCN normalized per nanogram of DNA in each location (*n* = 3). *Gray bars* represent the mean GCN normalized per milliliter of sample (*n* = 3). *Error bars* indicate standard deviations. Means with *different letters* indicate statistical significance between watershed sites at the 0.05 level

Quantitative PCR and FCM are powerful culture-independent methods used to quantitate microbial fractions or organisms in a variety of environments. Limitations exist for both approaches in terms of resolution, technical difficulty, variance, and dynamic range [84]. Microbial eukaryotes captured by the filtration system ranged between 1 to 105 μm in size, but in this study, a size cutoff of 5 μm was used for the larger organisms, suggesting that a significant portion of the microbial eukaryotes would have not been detected by the FCM. Another major constraint of FCM is the difficulty in designing a compatible dye or target-specific antigen for a specific target such as *E.coli* or T4-like myoviruses. In contrast, qPCR targeting specific genes are much simpler to design and implement. In this study, copy numbers of β-tubulin, 16S rRNA, *uidA*, and *g23* genes were estimated using qPCR (Fig. 1). Due to inaccuracies of DNA measurement by spectrophotometric methods, especially in the presence of inhibitors and contaminants, the GCNs reported in this study rely upon fluorometric measurements using the Qubit instrument (Table S4). As β-tubulin and 16S rRNA genes are multicopy genes, average factors of 1.93 (β-tubulin for eukaryotes) [85, 86] and 4.3 (16S rRNA genes for bacteria) [87] were used to normalize GCNs per nanogram and milliliter sample. The *uidA* gene is a single copy gene that encodes ß-D-glucuronidase in *E. coli* [88]. Quantitation of major capsid gene fragments for T4-like bacteriophages (*g23*) was conducted using viral DNA template with no random amplification step. Although the primer sets used to quantify GCNs were specific for these microbial fractions (Table 3), and non-specific amplification was not detected, PCR efficiency was low (~54 %) for β-tubulin and *g23*. This efficiency may have been improved by targeting a smaller DNA fragment (<300 bp); however, amplification of a shorter fragment of β-tubulin [86] was not successful in our samples, and the hypervariable regions within *g23* preclude qPCR of a shorter fragment [20, 89].

**Table 3:** Relative abundance (%) of *E. coli* in watershed sites using amplicon and metagenome approaches

Watershed site	16S rRNA*	*cpn*60*	Bacterial metagenome*
UPL	0.71 (198854)	0.24 (5955)	0.19 (44463)
UDS	0.65 (205568)	0.15 (10674)	0.17 (70203)
AUP	3.95 (253363)	2.62 (26641)	1.92 (48059)
APL	0.94 (38376)	1.54 (43417)	1.43 (169295)
ADS	0.34 (86499)	0.43 (53794)	0.44 (29399)
PUP	1.69 (66825)	0.60 (8947)	0.06 (71837)
PDS	0.16 (320422)	0.02 (11374)	0.42 (68525)

Numbers in parentheses represent total number of reads post quality filtering

*Correlation coefficients: 16S rRNA and *cpn*60 (*p* value = 0.0104, *r*_s_ = 0.8726); *cpn*60 and bacterial metagenome (*p* value = 0.0018, *r*_s_ = 0.9374)

Estimates of 16S rRNA gene abundances (Fig. 1a) were similar to those detected in other aquatic environments [86, 90–93]. The concentrations of prokaryotic cells estimated using 16S rRNA gene copies and flow cytometry counts were not significantly correlated (*p* > 0.05). GCNs of the 16S rRNA gene per milliliter of sample were between 0.8 to 1.6 orders of magnitude higher compared to FCM counts. Overestimation of prokaryotes by 16S rRNA qPCR can be associated with the multicopy nature and intragenomic heterogeneity of 16S rRNA [94, 95]. Quantitation of *E. coli* using the *uidA* gene (Fig. 1b) indicated that *E. coli* represented only 0.074 and 0.025 % of the biomass (GCN/ng DNA) and volume (GCN/mL of sample), respectively, within the bacterial fraction.

Estimates of eukaryote abundance using the β-tubulin gene indicated a range between 10^2^ to 10^4^ organisms across the watershed sites studied (Fig. 1c), which is comparable to previous studies [86, 96–98]. Quantitation of *g23* estimated the presence of between 10^2^ and 10^4^ T4-like bacteriophages per nanogram of DNA and per milliliter of sample (Fig. 1d). As the *g23* gene is found in T4-type bacteriophages, these numbers represent only a small fraction of the entire viral community that infects bacteria and an even smaller proportion of the entire viral community. While a comparison between *g23* and other viral groups is difficult, quantitation results via qPCR for other DNA viral groups such as adenovirus and JC polyomavirus in other freshwater ecosystems [99] were within the same order of magnitude as our samples.

Variables such as total coliform and *E. coli* counts, specific conductivity, total dissolved solids, salinity, turbidity, dissolved chloride, ammonia, orthophosphates, nitrites, and nitrates were found to be significantly correlated with *g23* GCNs/mL (*p* ≤ 0.0280, data not shown). As these variables increased, the abundance of major capsid genes increased as well. This finding suggests that these environmental variables and enterobacteria may have influence on the viral population, particularly T4-like myoviruses as previously reported in other freshwater ecosystems [100]. No other significant correlations were detected between the other two microbial fractions and environmental parameters.

The ratios of GCNs between β-tubulin, 16S rRNA, *uidA*, and *g23* were also determined. A ratio of 1:10^3^ was determined for comparison between *E. coli* (*uidA*) and total bacteria (16S rRNA gene). A further comparison between total *E. coli* counts (*uidA* gene fragments) and culturable *E. coli* cells (Colilert) indicated a difference of two to three orders of magnitude higher for quantitation using the *uidA* gene. This variation between culture-based and molecular-based *E. coli* assays has been previously reported [101]. The ratio of both β-tubulin and *g23* GCNs to 16S rRNA GCNs was on average 1:100, similar to other aquatic ecosystems [86, 97, 102–104]. As ecological relationships in aquatic environments are complex, the ratios described here only represent early insights into the microbial community interactions of these watershed locations.

### Microbial community structure in watersheds

Although a small number of samples were analyzed using the Bray-Curtis metric, the protected downstream (PDS) site stood apart from all sites (Additional file 1: Figure S5). Biofilms present in the 8.8-km pipe (Table 2) may have affected the microbial community composition resulting in a distinctive pattern for PDS compared to other watershed locations. The microbial communities not impacted by urban or agricultural activities, such as PUP and AUP, were more similar to one another (Additional file 1: Figure S5). Additional file 1: Tables S5 and S6 summarize read lengths and CG-contents of amplicon and metagenomic libraries, respectively.

Most of the rarefaction curves in the metagenomic and amplicon libraries plateaued (with singleton sequences removed), suggesting that most of the diversity within the eukaryotic, bacterial, and viral communities was captured. Diversity and richness indices were also calculated (Additional file 1: Tables S7 and S8). Although rarefaction curves approached an asymptote, there were differences in terms of community structure in each target fraction across the watershed sites. For instance, APL had the greatest diversity and richness values for bacteria based on the metagenomic data (Additional file 1: Figure S4 and Table S8). However, this community pattern changed when 16S rRNA and *cpn*60 amplicons were used (Additional file 1: Figure S3 and Table S7). These differences reflect the biases in PCR amplification, multicopy gene abundance, variation in genome sizes, library preparation, and normalization methods [105–107]. Thus, comparisons can only be made between samples prepared and analyzed using the same methods. In the present study, our main goal was to demonstrate the utility of a size fractionation method to capture most of the diversity and richness of the microbial community present in these watersheds. Percentage of significant hits assigned to each amplicon and bacterial and viral metagenomes are listed in Additional file 1: Table S9 and further detailed in Additional file 2.

### Characterization of microbial eukaryotic communities in watersheds using 18S rRNA and ITS amplicons

**Fig. 2:**
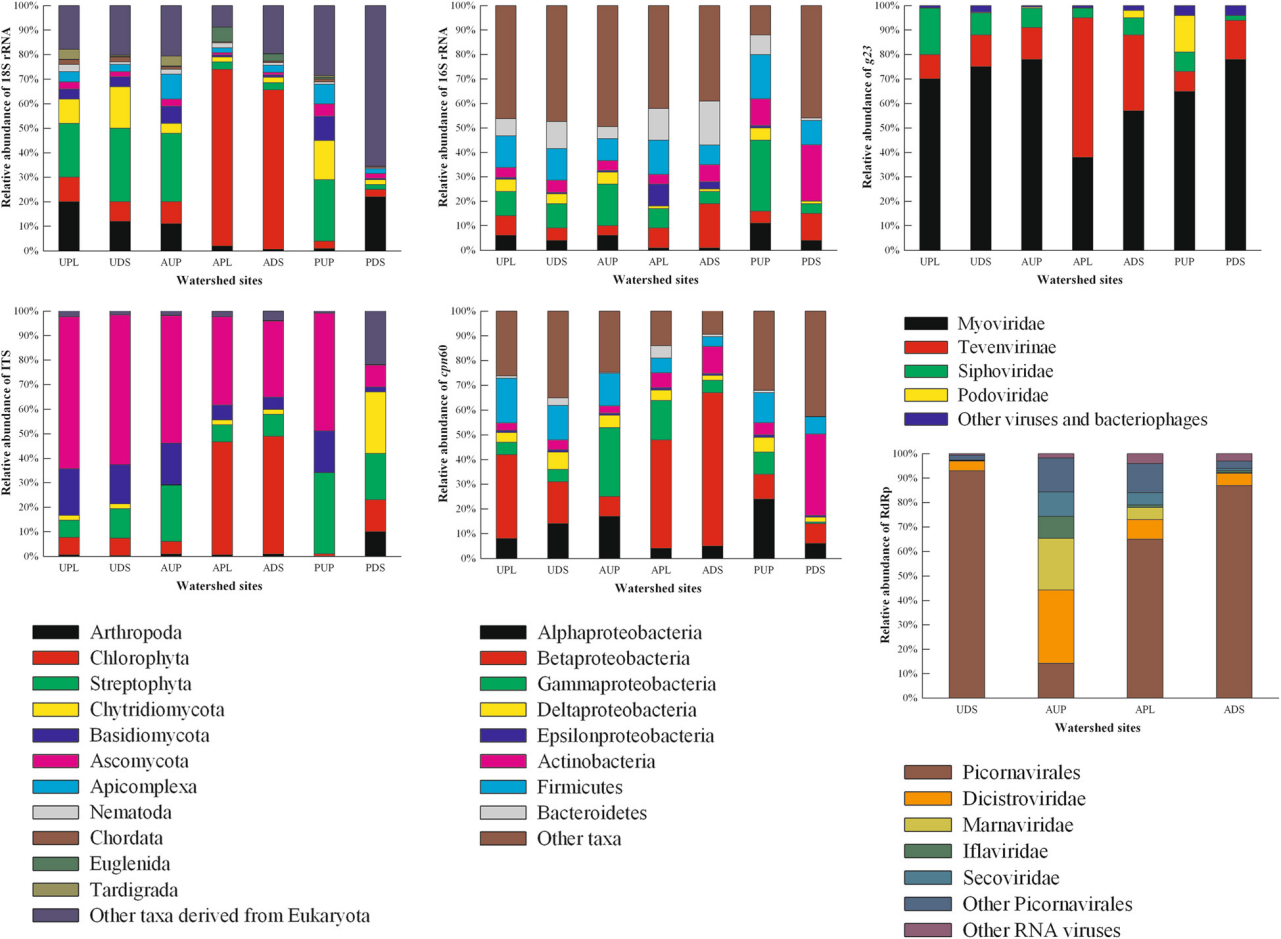
Relative abundance of eukaryotic (18S rRNA and ITS), bacterial (16S rRNA and cpn60), and viral communities (g23 and RdRp) identified in watershed sites. *UPL* urban polluted, *UDS* urban downstream, *AUP* agricultural upstream site, *APL* agricultural polluted, *ADS* agricultural downstream, *PUP* protected upstream, *PDS* protected downstream

Major taxa identified by 18S rRNA sequencing included Chlorophyta, Arthropoda, Streptophyta, Chytridiomycota, Apicomplexa, Nematoda, and Chordata (Fig. 2) while internal transcribed spacer (ITS) sequencing identified major groups such as algae, Chlorophyta, and two fungal phyla, Basidiomcycota and Ascomycota. Streptophyta were detected in the same abundance (16 %) with both 18S rRNA and ITS targets across the sites (Fig. 2). Other taxa detected by ITS sequencing had a lower representation than the same groups measured by 18S rRNA. Approximately 20 % of the 18S rRNA sequences were assigned as unclassified groups within Eukaryota, with less than 1 % related to fungi. In contrast, ~4.9 % of the ITS sequences were unclassified eukaryotic groups with 50 % of the taxa associated to unclassified fungi. Biases in amplification and sequencing (including sequencing platform) are well described for the 18S rRNA and ITS targets [108–111] and for other microbial targets as well [112]. Overall, Chlorophyta were a dominant group using both 18S rRNA and ITS sequencing in the agricultural impacted sites (APL and ADS), while Streptophyta appeared more abundant in urban sites with the 18S rRNA sequencing approach. Ascomycota were observed to be dominant in urban sites (UPL and UDS) with the ITS approach compared to other watershed locations. In this study, both 18S rRNA and ITS sequencing indicated differences among eukaryotic groups within each watershed.

### Characterization of bacterial communities in watersheds

**Fig. 3:**
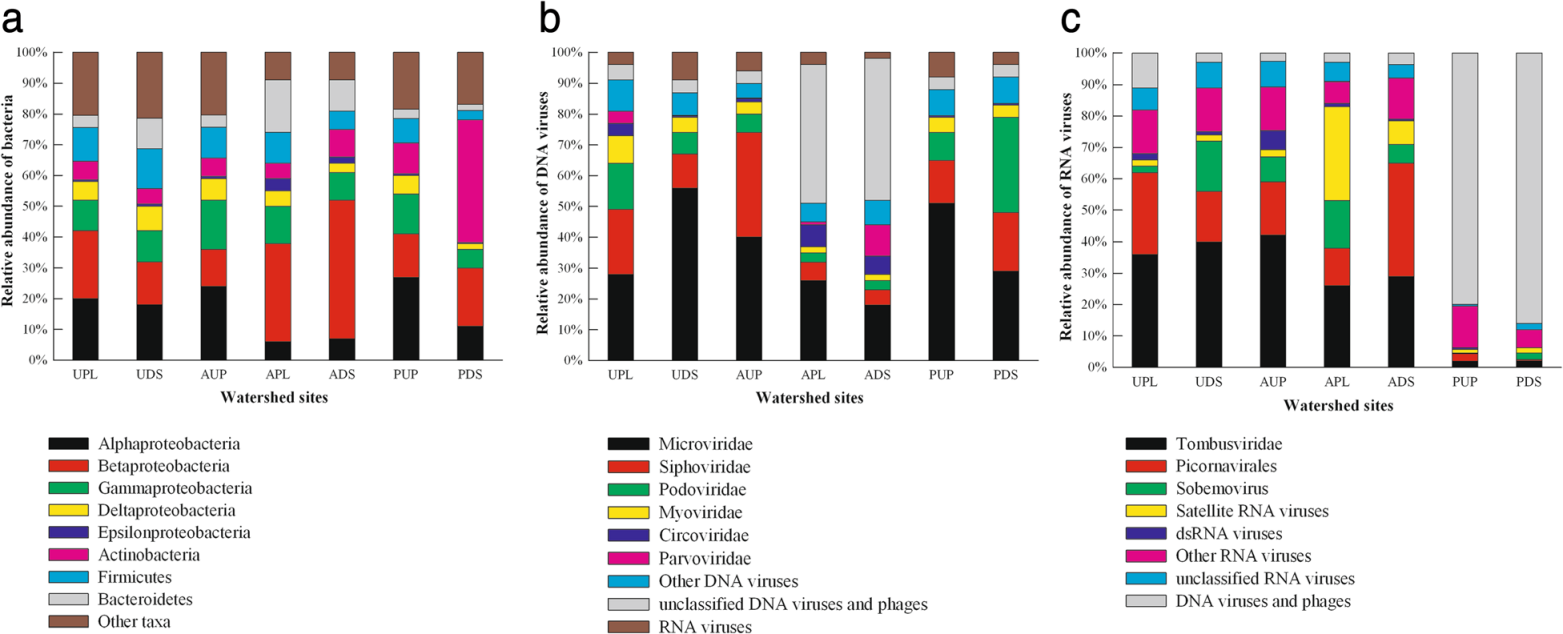
Relative abundance of bacterial and viral communities characterized using a metagenomic approach in watershed sites. *UPL* urban polluted, *UDS* urban downstream, *AUP* agricultural upstream site, *APL* agricultural polluted, *ADS* agricultural downstream, *PUP* protected upstream, *PDS* protected downstream. Taxonomic classes of bacteria (**a**), taxonomic groups of DNA viruses (**b**), and taxonomic groups of RNA viruses (**c**)

Abundant phyla of bacteria identified in the watershed samples included the four predominant phyla in the human and wildlife gut and in sediments and soil [113–115]: Proteobacteria (Alpha-, Beta-, Gamma-, Delta-, and Epsilonproteobacteria), Actinobacteria, Firmicutes, and Bacteroidetes (Fig. 3). Betaproteobacteria was the most abundant class of the Proteobacteria and made up ~17, 35, and 11 % of the bacterial community in amplicon and metagenome libraries from urban, agricultural impacted, and protected watersheds, respectively. While this class is a common feature reported in freshwater environments [116], significant differences (*p* ≤ 0.0184) were detected between agricultural and urban sites and protected watersheds.

Of the Firmicutes, order Clostridiales made up 73 % in agricultural impacted watersheds, 68 % in urban impacted watersheds, and 53 % in protected watersheds. Across watersheds, approximately 60 % of Clostridiales belonged to Clostridiaceae family. Other families accounted for ~30 % of the Clostridiales and included Ruminococcaceae, Peptococcaceae, Eubacteriaceae, Lachnospiraceae, and Syntrophomonadaceae. In small percentages (≤3 %), Heliobacteriaceae, Peptostreptococcaceae, and Clostridiales families XI, XVII, and XVIII were also observed. Interestingly, in agricultural- and urban-impacted watersheds, families such Ruminococcaceae and Lachnospiraceae were observed to be at least onefold higher than in protected sites (~1.5 %), while members of Peptostreptococcaceae were completely absent from protected watersheds. Genera of these families have been reported associated to fecal and sewage infrastructures [12, 117]. Another major order in Firmicutes was Bacillales; estimates of 13, 16, and 32 % were observed in agricultural, urban, and protected watersheds, respectively. The majority of Bacillales belonged to Bacillaceae (~60 %) and Paenibacillaceae (~20 %). This abundance of Bacillales in freshwaters is supported by the oxygenated and non-impacted nature of protected watersheds compared to agricultural or urban watersheds [118].

Both amplicon and metagenomic sequencing also revealed that Actinobacteria had high abundance in protected watersheds (~20.3 %), particularly PDS (~32 %) compared to urban (4.5 %) and agricultural (7.0 %) influenced watersheds (Figs. 2 and 3). Although Actinobacteria has routinely been reported as soil bacteria, its occurrence in freshwater and mainly in pristine water bodies could be attributed to soil pH [119, 120]. Within this phyla, order Actinomycetales made up >84 % of Actinobacteria in all watersheds. On average, 16 % of Actinomycetales pertained to Streptomycetaceae in urban and protected watershed, while in agricultural sites, this family represented 5 % or less. A high abundance of bacterial groups within Streptomycetaceae has been associated to the production of geosmin and 2-methylisoborneol, which impact water quality [121, 122]. Other orders included Solirubrobacterales, Rubrobacterales, Acidimicrobiales, Coriobacteriales, and Bifidobacteriales, which made up less than 10 % of the Actinobacterial community in watersheds. Within order Bifidobacteriales, Bifidobacteriaceae (ubiquitous inhabitants of the gastrointestinal tract of mammals) were dominant in urban impacted sites (~3.4 %) compared to agricultural (~1.2 %) or protected (~0.4 %) watersheds.

Although only seven environmental samples were analyzed in this study, a positive correlation (*p* ≤ 0.05) was identified between abundances of Epsilonproteobacteria and Bacteroidetes across watershed sites. Abundances for both classes were found to be higher (*p* ≤ 0.0476) in agricultural and urban sites compared to protected watersheds. Both of these classes of bacteria have been reported to play an important role in coastal and ocean waters by reducing sulfidic conditions in sediments, anoxic water columns, and oxygen minimum zones [123, 124]. These shifts in bacterial population structure may represent an ecological succession in community structure as a response to the conditions in the impacted watersheds [123].

While relative abundances were used to compensate for library variability and read numbers for each library, clear differences were observed among taxa within the same fraction. For instance, a comparison of taxa abundances generated by the bacterial amplicon and metagenomic sequencing shows that the 16S rRNA amplicon data predict lower abundance of certain bacterial groups, such as Alpha- and Betaproteobacteria, relative to *cpn*60 and metagenome sequencing (Figs. 2 and 3). In contrast, *cpn*60 sequencing predicted a lower Bacteroidetes abundance compared to 16S rRNA and metagenome sequencing. Moreover, classes such as Planctomycetacia, Chloroflexi, and Verrucomicrobiae represented ~50 % of other taxa using *cpn*60 sequencing, while 16S rRNA sequencing identified the same classes in approximately ≤1 % among other taxa in most samples.

*E. coli*, a fecal indicator organism, was further compared with other taxa using amplicon and metagenome sequencing. *E. coli* abundance is difficult to measure using 16S rRNA due to the high 16S sequence similarity among genera in the *Enterobacteriaceae* family. However, significant correlations were detected across sites for *E. coli* abundance between 16S rRNA and *cpn*60 amplicons (*p* = 0.0104) and *cpn*60 and metagenomic libraries (*p* = 0.0018) (Table 3). Metagenome data revealed other taxa such as *Polynucleobacter*, *Arcobacter*, *Methylotenera*, *Flavobacterium*, *Pseudomonas*, and *Bacteroides* were found in higher relative abundance compared to *E. coli* in urban and agricultural influenced watersheds. Some of these alternative indicators have been previously reported in urban-impacted watersheds [7]. *Polynucleobacter* was abundant in amplicon and bacterial metagenome of impaired watersheds. The ubiquity of this genus has been reported in freshwater lakes and rivers associated to ecological diversification [125, 126] and may be an indicator of impacted environments.

### Characterization of viruses in watersheds

For DNA virus metagenomic libraries, the rate of taxonomic classification to viral taxa was between 7.81 % (UPL) and 18.89 % (PDS), while for RNA viruses, this number ranged from 1.2 % (UPL) to 17.06 % (APL) (Additional file 1: Table S6). Taxonomic analysis revealed six major groups of DNA viruses and four major groups of RNA viruses (Fig. 3c), with *Microviridae* and *Tombusviridae* being the most well represented groups of DNA and RNA viruses, respectively. The majority of assigned viral DNA sequences (82 %) corresponded to viruses or phages infecting bacteria such as *Microviridae*, *Siphoviridae*, *Myoviridae*, and unclassified DNA viruses and phages, while 64 % of assigned RNA virus sequences were identified as algal and plant pathogens such as *Tombusviridae*, *Picornavirales*, *Sobemovirus*, and satellite RNA viruses. These families and groups have previously been reported as abundant in other freshwater ecosystems that are rich in organics [73, 127], suggesting trophic status or productivity may influence viral hosts and therefore affect viral community structure. In this context, the remaining groups of DNA viruses included *Circoviridae* and *Parvoviridae*, which are known to contain plant and animal viruses [81, 128, 129].

Despite efforts to eliminate DNA from the viral RNA fraction, a significant number of DNA virus sequences were detected in the viral RNA fraction across all sites. These sequences were identified as belonging to groups such as *Caudovirales*, *Circoviridae*, *Microviridae*, classified and unclassified phages infecting prokaryotes, and unclassified ssDNA viruses. A relatively small percentage (~5 %) of RNA viruses were identified in the viral DNA fraction. Other researchers have also reported DNA and RNA viruses in the opposite fractions [79, 131]. A probable explanation for these observations may be related to DNA and RNA reverse transcribing viruses combined with representational deficiencies in existing viral database [11, 130, 131].

Sequencing of *g23* revealed ~92 % of sequences matched the *Myoviridae* family including *Enterobacteria* phages, T4-like viruses, and *Klebsiella* phages. Moreover, the relative abundance of RdRp sequences indicated *Picornavirales* as the most abundant group across sites with JP-B-like viruses accounting for ~90 % of the *Picornavirales* sequences detected. RdRp amplicons in AUP revealed a more homogeneous distribution of picorna-like viruses compared to the other watersheds (Fig. 2). Other members of this picorna-like superfamily were *Dicistroviridae*, *Marnaviridae*, *Iflaviridae*, and *Secoviridae*, which have been reported as insect and algae-infecting viruses [11, 132]. Compared to the other microbial fractions, a majority of metagenome viral sequences remained unclassified suggesting that a larger, more complete viral database is needed in order to identify these unknown viruses. These results are largely congruent with other studies in aquatic ecosystems [27, 81, 133, 134], which have also reported a majority of sequences to have unknown origins.

### Assessment of community structure among microbial fractions

Sequencing analysis revealed the presence of larger and smaller organisms within each size fraction, but this percentage was minimal within the bacterial and viral DNA fractions. From the bacterial fraction sequencing reads, 96.3 % were associated with bacteria and the remainder classified as Archaea (2.2 %), Eukaryotes (1.3 %), and viruses comprised less than 0.2 % (not PhiX sensu lato). Ribosomal RNA was detected in viral RNA fraction with percentages ranging from 5.3 to 24.5 %. Ribosomal RNA in the viral DNA fraction was found in less than 1 % of the reads. These ribosomal RNAs belonged to bacterial and eukaryotic groups also found in the bacterial metagenome. We hypothesize that free ribosomes from lysed cells (including from the viral lytic cycle) could persist in the environment and would pass through all the filters. Although RNA levels were low or below the detection limit in the viral fraction, the random amplification process could have amplified this material [135]. Also, eluates were not treated with RNase due to the potential degradation of some RNA viruses [81, 136, 137].

Additionally, the Enterobacteria phage PhiX sensu lato, a control used during sequencing, was detected among the viral metagenomic and amplicon libraries, but not in bacterial metagenomic libraries. PhiX contamination averaged ~4 % in the *g23*, RdRp, and viral RNA libraries and ~32 % in the viral DNA libraries. This adapter-ligated control was identified in ~0.34 % of the reads in other non-viral amplicon libraries such as ribosomal RNA (18S and 16S rRNA genes) and ITS. Mock communities (bacterial and viral) and amplicons (16S rRNA, *g23*, and RdRp) used as sequencing controls were further analyzed for possible PhiX contamination. Similar to bacterial libraries from environmental samples, no PhiX sensu lato was found in mock bacterial libraries. In viral and amplicon control libraries, less than 1 % of the total reads were assigned to PhiX. A probable explanation may be related to random cluster dispersal that may result in cluster overlap during Illumina sequencing [138]. The high percentage of PhiX in the viral DNA libraries suggests that PhiX (a coliphage) was indeed infecting bacteria such *E. coli* in these samples [139, 140]. A correlation analysis performed between the abundance of PhiX observed in samples and the relative sequence abundance of *E. coli* (Table 3) did not detect any significant differences. While the occurrence PhiX sensu lato in viral DNA reads may reflect their actual presence in the environment, reads associated to this bacteriophage were excluded from the results described here as we cannot determine how much could be due to contamination. An alternative approach would have been to skip the addition of PhiX to the libraries or use a different adapter-ligated control in order to corroborate for the presence of these coliphages in the samples.

### Gene function analysis and composition

**Fig. 4:**
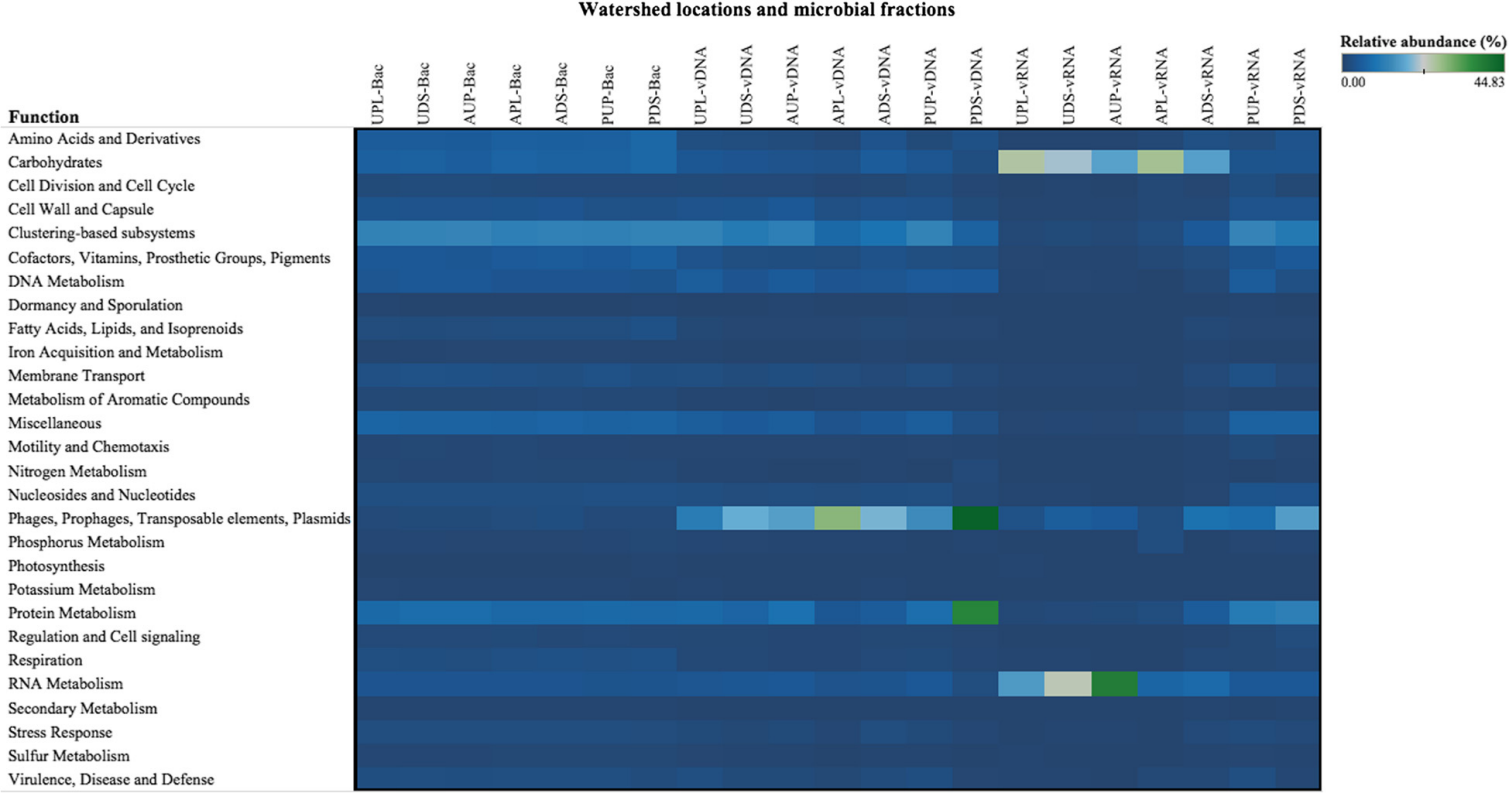
Heat map of functional categories for bacterial, viral DNA, and viral RNA metagenomes across watershed locations. *UPL* urban polluted, *UDS* urban downstream, *AUP* agricultural upstream site, *APL* agricultural polluted, *ADS* agricultural downstream, *PUP* protected upstream, *PDS* protected downstream, *Bac* bacteria, *vDNA* viral DNA, *vRNA* viral RNA

Besides improving to taxonomic analysis, metagenomic sequencing has the advantage of enabling the analysis of the functional gene content of the microbial community. Comparison of gene function abundances in the bacterial fraction using SEED subsystem classifications revealed no major differences across sites (Fig. 4). Genes with unknown function that are hypothesized to have similar functions based on their locations across genomes (clustering-based subsystems) made up most of the bacterial annotations (~12.23 %), followed by genes involved in protein metabolism (~7.35 %). Phage-associated genes, including those coding for capsid proteins and terminases, were the most abundant functional annotations in the viral DNA fraction (Fig. 4). These genes are major components of the phage DNA packaging machinery [141, 142]. Notably, in the RNA fraction, retron-type reverse transcriptase genes (RNA metabolism category) were found in higher relative abundance in AUP and urban sites compared to other sites (Fig. 4). This reverse flow of genes is considered a fingerprint of viral replication in any system [143].

As parasites of their hosts, viruses enter and hijack natural cellular process [144]. Universal glycoproteins such as GTPases made up significant proportions of the protein metabolism within the viral DNA fraction. Higher abundance of genes involved in carbohydrate metabolism (mainly ribokinases) was observed within the viral RNA fraction. The acquisition of cellular genes may reflect host-defense mechanisms against viruses and utilization of the host machinery for the viruses to replicate. Consistent with the findings from the taxonomic characterization, where DNA viruses were found in the viral RNA fraction, analysis of putative functions revealed DNA virus-associated genes (encoding for phage capsids and scaffolds) in some of the viral RNA samples.

Overall, a wide variety of taxa and genes were predicted using amplicon and metagenomic approaches. As discussed above, this variability in microbial communities can be attributed to a combination of the survey methods, organism/particle sizes, anthropogenic affects, and environmental conditions.

## Conclusion

We have developed a method for separating different sized microorganisms (eukaryotes, bacteria, and viruses) from water samples and comprehensive characterization of the water microbiota. So far, few studies have attempted to characterize multiple microbial domains from the same environmental sample due to technical challenges, the effort required, and the cost. The use of a systematic size fractionation approach enabled enrichment of a particular fraction and minimized noise during metagenomic sequencing from larger fractions to the smaller ones. While multiple associations were observed between classes and sites, we have highlighted only the most representative findings as the focus was to develop a robust methodology for extensive watershed microbiome analysis. The use of metagenomics to characterize microbial communities provides insights not only into the wider range of microbial eukaryotic, bacteria, and viral taxa present in watersheds but also for further analysis of the functional gene complement in these microbial communities. Note that a large proportion of viral amplicon and metagenomic sequence data remained unassigned, supporting the need for further study of viral diversity and development of viral sequence databases for reference-based analysis. A year-long, large-scale watershed metagenomic project (http://www.ncbi.nlm.nih.gov/bioproject/287840) has employed the methods described here, and this report describes the early findings from this study. This larger project aims to further characterize profiles of microbial eukaryotes, bacteria, and viruses, combined with physical, chemical, and biological indicator data, in multiple watersheds in British Columbia with the ultimate goal of discovering new biomarkers to monitor water quality. We hope that the methods described here and the accompanying preliminary data will support other similar holistic analyses of water microbial communities and not only improve our understanding of the complexities of the water microbiome but also further our ability to protect our watersheds.

## Additional files

Additional file 1: Word document includes table of contents for supplemental results, discussion, and detailed methods. Supplemental tables (Tables S1–S9) and figures (Figures S1–S5) are also included. (DOCX 785 kb) Additional file 2: Excel file contains output data from MG-RAST, mPUMA, and MetaVir reported in this study. Singletons have been removed from the analysis. (XLSX 1941 kb)

## Abbreviations

*cpn*60: chaperonin-60
FCM: flow cytometry
GCN: gene copy number
ITS: internal transcribed spacer
*g23*: major capsid gene
qPCR: quantitative PCR
RdRp: RNA-dependent RNA polymerase
rRNA: ribosomal RNA
TFF: tangential flow filtration
