# Supplementary material for: A comprehensive method for amplicon-based and metagenomic characterization of viruses, bacteria, and eukaryotes in freshwater samples
Source: Microbiome. 2016 Jul 11;4(1):20. doi: 10.1186/s40168-016-0166-1 (PMC5011856; doi:10.1186/s40168-016-0166-1)
Supplement: Additional file 1: — Word document includes table of contents for supplemental results, discussion, and detailed methods. Supplemental tables (Tables S1–S9) and figures (Figures S1–S5) are also included. (DOCX 785 kb) [file 40168_2016_166_MOESM1_ESM.docx]

**Table of contents**

Supplemental tables ii

Table S1. Description of primer sets used in PCR and qPCR of adenoviruses and enteroviruses ii

Table S2. Water quality parameters of watershed sites iii

Table S3. Flow cytometry counts of virus-like particles and bacterial cells per mL of sample iv

Table S4. Assessment of the quality and quantity of nucleic acids extracted from watershed sites v

Table S5. Summary statistics of amplicon sequences post quality filtering vi

Table S6. Summary statistics of bacterial and viral DNA and RNA sequences post quality-filtering vii

Table S7. Diversity and richness indices calculated for assigned amplicon sequences from watershed samples viii

Table S8. Diversity and richness indices calculated for assigned metagenome sequences from watershed samples ix

Table S9. Percentage of significant hits assigned to eukaryotes, bacteria, viruses in watershed sites using amplicon and metagenomic sequencing x

Supplemental figures xi

Fig. S1 Quantitation of adenoviruses (A) and enteroviruses (B) using quantitative PCR and flow cytometry xi

Fig. S2 Relationship between quantitative PCR and flow cytometry approaches to quantify viral particles of adenoviruses and enteroviruses xiii

Fig. S3 Rarefaction analysis of amplicon sequences: 18S rRNA, ITS, 16S rRNA, *cpn*60, *g23*, and RdRp observed in watershed sites xiv

Fig. S4 Rarefaction analysis of A) bacterial, B) viral DNA, and C) viral RNA communities observed in watershed sites xvi

Fig. S5 Principal coordinate analysis using Bray-Curtis dissimilarity measures (beta diversity) for metagenomes and amplicons in watershed locations xviii

**Supplemental tables**

**Table S1** Description of primer sets used in PCR and qPCR of adenoviruses and enteroviruses

| Target gene | Primer name | Primer sequences (5’🡪 3’) | Amplicon size (bp) | Reference |
| --- | --- | --- | --- | --- |
| hAdV hexon | Ad2-F  Ad2-R  Ad4-F  Ad4-R | CCAGGACGCCTCGGAGTA  AAACTTGTTATTCAGGCTGAAGTACGT  GGACAGGACGCTTCGGAGTA  CTTGTTCCCCAGACTGAAGTAGGT | 87 | [35] |
| UTR*^e^* | Verstrepen-F  Watzinger-R | CCCTGAATGCGGCTAATCC  ARATTGTCACCATAAGCAGCCA | 148 | [36]  [37] |

**Table S2** Water quality parameters of watershed sites

| Sites | T (°C) | DO (mg/L) | SC (μS/cm) | TDS (mg/L) | SAL (PSU) | pH | PRES (mmHg) | TURB (NTU) | TCO | ECO | DCH (mg/L) | NH_4_^3-^ (mg/L) | PO_4_^3-^ (mg/L) | NO_2_^-^ (mg/L) | NO_3_^-^ (mg/L) | Flow rate (m^3^/sec) |
| --- | --- | --- | --- | --- | --- | --- | --- | --- | --- | --- | --- | --- | --- | --- | --- | --- |
| UPL | 5.5 | 13.17 | 53.1 | 34.45 | 0.02 | 6.38 | 733.3 | 0.79 | 98 | <10 | 6.1 | 0.0055 | 0.0042 | 0.011 | 2.6498 | 0.06 |
| UDS | 6.0 | 12.65 | 99.5 | 64.35 | 0.05 | 7.07 | 744.5 | 1.51 | 1313 | 249 | 13 | 0.0092 | 0.0060 | 0.011 | 2.8428 | 0.29 |
| AUP | 4.3 | 12.46 | 90.3 | 58.5 | 0.04 | 7.21 | 748.6 | 1.76 | 10 | 10 | 0.9 | 0.0159 | 0.0027 | 0.003 | 3.5383 | 0.16 |
| APL | 7.5 | 4.02 | 283.9 | 184.6 | 0.14 | 6.85 | 758.6 | 18.87 | 2284 | 399 | 14 | 0.848 | 0.1264 | 0.16 | 6.7194 | 2.11 |
| ADS | 7.5 | 8.85 | 257.1 | 160.5 | 0.12 | 7.11 | 758.8 | 13.37 | 789 | 98 | 12 | 0.266 | 0.1436 | 0.07 | 8.9343 | 9.97 |
| PUP | 6.6 | 10.24 | 42.1 | 27.3 | 0.02 | 7.12 | 752.1 | 0.20 | 8.4 | <1 | 2.2 | 0.0117 | 0.0030 | 0.006 | 0.0297 | 0.6 |
| PDS | 6.1 | 11.48 | 40.2 | 26.0 | 0.02 | 6.76 | 743.2 | 0.31 | 10 | <1 | 2.4 | 0.0174 | 0.00095 | 0.0044 | 0.1021 | 1.01 |
| T = temperature; DO = dissolved oxygen; SC = specific conductivity; TDS = total dissolved solids; SAL = salinity; PRES = atmospheric pressure; TURB = turbidity; TCO = total coliform counts per 100 mL sample; ECO = *E. coli* counts per 100 mL sample; DCH = dissolved chloride. | | | | | | | | | | | | | | | | |

**Table S3** Flow cytometry counts of virus-like particles and bacterial cells per mL of sample. Numbers represent mean values (n=2)

| Watershed sites | Virus-like particles  in raw water | Virus-like particles  in viral concentrate | Concentration efficiency % | Bacterial cells  in raw water | Bacterial cells  in 0.2 μm filter | Concentration efficiency % | Filtrated  volume (L) |
| --- | --- | --- | --- | --- | --- | --- | --- |
| UPL | 5.03 x 10^6^ | 3.67 x 10^8^ | 86.1 | 1.55 x 10^5^ | 1.15 x 10^5^ | 74.2 | 37.5 |
| UDS | 7.09 x 10^6^ | 5.24 x 10^8^ | 81.6 | 2.07 x 10^5^ | 1.66 x 10^5^ | 80.4 | 37.1 |
| AUP | 1.60 x 10^6^ | 2.48 x 10^8^ | 149.7 | 7.52 x 10^4^ | 6.02 x 10^4^ | 80.0 | 39.4 |
| APL | 1.18 x 10^8^ | 2.76 x 10^8^ | 2.4 | 1.24 x 10^6^ | 1.24 x 10^6^ | 99.7 | 39.7 |
| ADS | 6.86 x 10^7^ | 3.34 x 10^8^ | 5.5 | 1.10 x 10^6^ | 1.10 x 10^6^ | 99.8 | 38.8 |
| PUP | 6.10 x 10^6^ | 3.98 x 10^8^ | 71.2 | 7.60 x 10^4^ | 7.38 x 10^4^ | 97.1 | 40.2 |
| PDS | 1.92 x 10^7^ | 9.60 x 10^8^ | 50.5 | 3.34 x 10^5^ | 3.28 x 10^5^ | 98.3 | 38.2 |

**Table S4** Assessment of the quality and quantity of nucleic acids extracted from watershed sites

| Watershed site | 1 μm-filter | | | 0.2 μm-filter | | | TFF (30 kDa) | | | | | |
| --- | --- | --- | --- | --- | --- | --- | --- | --- | --- | --- | --- | --- |
|  | Concentration ng DNA/mL sample* | A_260/280_ | A_260/230_ | Concentration ng DNA/mL sample* | A_260/280_ | A_260/230_ | Concentration ng DNA/mL sample* | A_260/280_ | A_260/230_ | Concentration ng RNA/mL sample* | A_260/280_ | A_260/230_ |
| UPL | 0.03 (0.003) | 1.7 | 0.5 | 0.06 (0.02) | 1.7 | 0.7 | 0.08 (0.01) | 1.5 | 0.6 | BDL | - | - |
| UDS | 0.02 (0.003) | 1.5 | 0.5 | 0.07 (0.03) | 1.7 | 0.8 | 0.05 (0.02) | 1.6 | 0.7 | BDL | - | - |
| AUP | 0.03 (0.02) | 1.7 | 1.0 | 0.02 (0.01) | 1.8 | 0.7 | 0.03 (0.01) | 1.7 | 0.8 | 0.02 (BDL) | 1.6 | 0.8 |
| APL | 1.15 (0.04) | 1.8 | 1.7 | 0.01 (0.01) | 1.7 | 0.9 | 0.03 (0.01) | 1.8 | 1.0 | 0.02 (0.004) | 1.9 | 1.0 |
| ADS | 0.84 (0.04) | 1.8 | 1.7 | 0.04 (0.02) | 1.8 | 2.1 | 0.10 (0.01) | 1.7 | 1.0 | 0.08 (0.004) | 1.7 | 1.0 |
| PUP | 0.02 (0.03) | 2.0 | 1.0 | 0.06 (0.05) | 1.9 | 1.1 | 0.09 (0.01) | 1.4 | 0.7 | BDL | - | - |
| PDS | 0.07 (0.05) | 1.8 | 1.1 | 0.13 (0.05) | 1.9 | 1.7 | 0.15 (0.04) | 1.6 | 0.9 | BDL | - | - |
| * Numbers represent concentration of nucleic acids using NanoDrop spectrophotometer, while values in parentheses represent concentration using Qubit DNA and RNA assay kits. BDL = below detection limit. | | | | | | | | | | | | |

**Table S5** Summary statistics of amplicon sequences post quality filtering

|  | 18S rRNA | | ITS | | 16S rRNA | | *cpn*60* | | *g23* | | RdRp | |
| --- | --- | --- | --- | --- | --- | --- | --- | --- | --- | --- | --- | --- |
| Sites | RL ± SD (bp) | GC ± SD (%) | RL ± SD (bp) | GC ± SD (%) | RL ± SD (bp) | GC ± SD (%) | RL ± SD (bp) | GC ± SD (%) | RL ± SD (bp) | GC ± SD (%) | RL ± SD (bp) | GC ± SD (%) |
| UPL | 193 ± 40 | 44 ± 6 | 220 ± 20 | 48 ± 6 | 214 ± 21 | 52 ± 4 | 474 ± 142 | 53 ± 6 | 221 ± 21 | 48 ± 6 | - | - |
| UDS | 203 ± 23 | 45 ± 5 | 217 ± 22 | 48 ± 7 | 210 ± 24 | 52 ± 4 | 475 ± 143 | 55 ± 6 | 224 ± 20 | 51 ± 5 | 165 ± 39 | 45 ± 4 |
| AUP | 201 ± 24 | 45 ± 5 | 212 ± 26 | 49 ± 7 | 212 ± 22 | 52 ± 4 | 486 ± 136 | 55 ± 5 | 221 ± 22 | 50 ± 6 | 206 ± 49 | 44 ± 6 |
| APL | 198 ± 23 | 43 ± 7 | 212 ± 24 | 48 ± 8 | 206 ± 25 | 50 ± 4 | 492 ± 131 | 53 ± 5 | 211 ± 25 | 42 ± 7 | 211 ± 25 | 42 ± 7 |
| ADS | 201 ± 23 | 45 ± 8 | 207 ± 29 | 49 ± 8 | 210 ± 21 | 51 ± 4 | 491 ± 133 | 54 ± 2 | 217 ± 22 | 51 ± 8 | 211 ± 27 | 41 ± 5 |
| PUP | 232 ± 10 | 45 ± 4 | 247 ± 7 | 49 ± 7 | 242 ± 8 | 52 ± 4 | 475 ± 143 | 56 ± 6 | 243 ± 11 | 47 ± 5 | - | - |
| PDS | 231 ± 4 | 46 ± 4 | 248 ± 5 | 48 ± 9 | 236 ± 8 | 54 ± 3 | 483 ± 129 | 53 ± 5 | 232 ± 9 | 51 ± 5 | - | - |
| RL: read length; SD: standard deviation  * *cpn*60 amplicons were sequenced on a Roche 454 Genome Sequencer FLX Titanium | | | | | | | | | | | | |

**Table S6** Summary statistics of bacterial and viral DNA and RNA sequences post quality filtering

| Sites | Bacteria | | Viral DNA | | Viral RNA | |
| --- | --- | --- | --- | --- | --- | --- |
|  | RL ± SD (bp) | GC ± SD (%) | RL ± SD (bp) | GC ± SD (%) | RL ± SD (bp) | GC ± SD (%) |
| UPL | 237 ± 45 | 51 ± 10 | 172 ± 54 | 49 ± 8 | 169 ± 50 | 48 ± 6 |
| UDS | 241 ± 44 | 51 ± 10 | 189 ± 59 | 46 ± 8 | 188 ± 52 | 46 ± 6 |
| AUP | 241 ± 44 | 52 ± 10 | 200 ± 58 | 46 ± 8 | 202 ± 51 | 44 ± 6 |
| APL | 236 ± 44 | 49 ± 10 | 191 ± 54 | 44 ± 8 | 202 ± 49 | 44 ± 7 |
| ADS | 232 ± 46 | 49 ± 10 | 196 ± 58 | 45 ± 8 | 207 ± 49 | 42 ± 7 |
| PUP | 193 ± 59 | 52 ± 12 | 199 ± 58 | 46 ± 8 | 171 ± 50 | 41 ± 8 |
| PDS | 240 ± 45 | 51 ± 9 | 197 ± 55 | 42 ± 9 | 182 ± 50 | 37 ± 8 |
| RL: read length; SD: standard deviation | | | | | | |

| Sites | 18S rRNA | | | ITS | | | 16S rRNA | | | *cpn*60 | | | *g23* | | | RdRp | | |
| --- | --- | --- | --- | --- | --- | --- | --- | --- | --- | --- | --- | --- | --- | --- | --- | --- | --- | --- |
|  | S | SW | C1 | S | SW | C1 | S | SW | C1 | S | SW | C1 | S | SW | C1 | S | SW | C1 |
| UPL | 0.93 | 3.68 | 596.74 | 0.95 | 4.18 | 900.09 | 0.73 | 2.57 | 1143.02 | 0.95 | 5.73 | 2922.38 | 0.87 | 2.31 | 536.77 | - | - | - |
| UDS | 0.92 | 3.45 | 760.12 | 0.95 | 4.07 | 750.19 | 0.81 | 3.11 | 1476.14 | 0.99 | 7.06 | 3425.38 | 0.90 | 2.87 | 565.27 | 0.16 | 0.54 | 145.5 |
| AUP | 0.92 | 3.57 | 748.10 | 0.95 | 4.04 | 1355.74 | 0.72 | 2.61 | 1327.52 | 0.96 | 6.60 | 3880.12 | 0.80 | 2.40 | 756.91 | 0.89 | 2.99 | 477.92 |
| APL | 0.86 | 2.69 | 462.01 | 0.81 | 2.72 | 687.20 | 0.70 | 2.59 | 873.12 | 0.96 | 4.64 | 1907.35 | 0.91 | 2.95 | 160.77 | 0.57 | 1.72 | 165.89 |
| ADS | 0.90 | 3.19 | 1017.84 | 0.86 | 3.08 | 984.74 | 0.69 | 2.43 | 1096.14 | 0.86 | 3.77 | 729.81 | 0.89 | 3.0 | 1274.4 | 0.26 | 0.9 | 197.04 |
| PUP | 0.87 | 3.19 | 2177.88 | 0.94 | 3.55 | 1251.23 | 0.74 | 2.73 | 1283.20 | 0.99 | 7.47 | 4253.51 | 0.87 | 2.30 | 27.6 | - | - | - |
| PDS | 0.90 | 3.14 | 1353.88 | 0.90 | 2.92 | 615.11 | 0.73 | 2.37 | 927.08 | 0.86 | 3.01 | 179 | 0.66 | 1.69 | 373 | - | - | - |
| S = Simpson’s index of diversity  SW = Shannon-Weaver diversity index  C1 = Chao1 richness estimator | | | | | | | | | | | | | | | | | | |

**Table S7** Diversity and richness indices calculated for annotated amplicon sequences from watershed samples

**Table S8** Diversity and richness indices calculated for assigned metagenomic sequences from watershed samples

| Sites | Bacteria | | | DNA viruses | | | RNA viruses | | |
| --- | --- | --- | --- | --- | --- | --- | --- | --- | --- |
|  | S | SW | C1 | S | SW | C1 | S | SW | C1 |
| UPL | 0.89 | 3.99 | 1452.86 | 0.99 | 5.98 | 1160.54 | 0.98 | 4.88 | 611.33 |
| UDS | 0.89 | 4.23 | 1493.79 | 0.99 | 6.33 | 1346.54 | 0.98 | 4.87 | 1699.02 |
| AUP | 0.88 | 4.02 | 1362.51 | 0.90 | 4.88 | 1397.07 | 0.98 | 5.05 | 2080.57 |
| APL | 0.95 | 4.52 | 1871.86 | 0.99 | 6.25 | 1508.87 | 0.96 | 4.07 | 253.94 |
| ADS | 0.94 | 3.97 | 1143.45 | 0.99 | 6.08 | 1315.85 | 0.96 | 4.24 | 1618.25 |
| PUP | 0.91 | 4.15 | 1518.07 | 0.99 | 6.10 | 1271.42 | 0.99 | 5.94 | 1477.68 |
| PDS | 0.91 | 3.81 | 1218.16 | 0.84 | 3.63 | 1369.30 | 0.98 | 5.09 | 1157.09 |
| S = Simpson’s index of diversity  SW = Shannon-Weaver diversity index  C1 = Chao1 richness estimator | | | | | | | | | |

**Table S9** Percentage of significant hits assigned to eukaryotes, bacteria, viruses in watershed sites using amplicon and metagenomic sequencing

| Sites | 18S^a^ | ITS^a^ | Bacteria (metagenome)^a^ | 16S^a^ | *cpn*60^b^ | Viral DNA (metagenome)^c^ | *g23*^c^ | Viral RNA (metagenome)^c^ | RdRp^c^ |
| --- | --- | --- | --- | --- | --- | --- | --- | --- | --- |
| UPL | 98.9 | 99.4 | 95.3 | 89.6 | 99.2 | 7.8 | 91.5 | 1.2 | - |
| UDS | 99.6 | 99.5 | 94.9 | 90.4 | 99 | 9.2 | 96.9 | 7.8 | 58 |
| AUP | 99.5 | 97.9 | 95.7 | 88.5 | 99 | 13.4 | 93.6 | 7.7 | 21 |
| APL | 98 | 85.7 | 96.3 | 96 | 99.5 | 12.9 | 99.5 | 17.1 | 56.3 |
| ADS | 94.4 | 79 | 96.8 | 95.3 | 99.2 | 13.1 | 91.5 | 16.1 | 80.3 |
| PUP | 99.6 | 97.8 | 96.7 | 86.4 | 99.3 | 8.7 | 50 | 8.7 | - |
| PDS | 99.9 | 95.1 | 98.1 | 93.3 | 99.6 | 18.9 | 87.8 | 15.1 | - |
| ^a^ MG-RAST; ^b^ mPUMA; ^c^ Metavir | | | | | | | | | |

**Supplemental Figures**


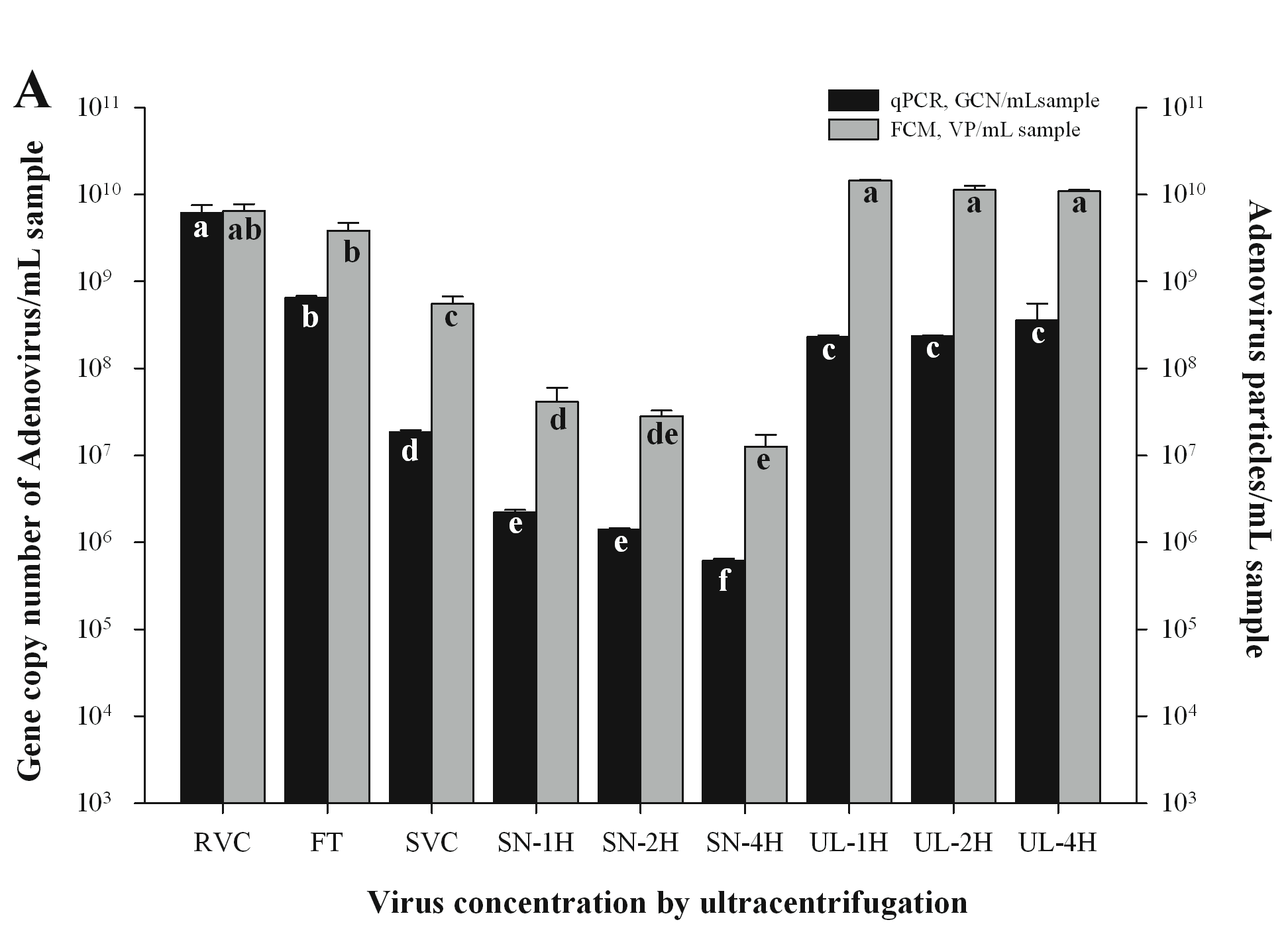


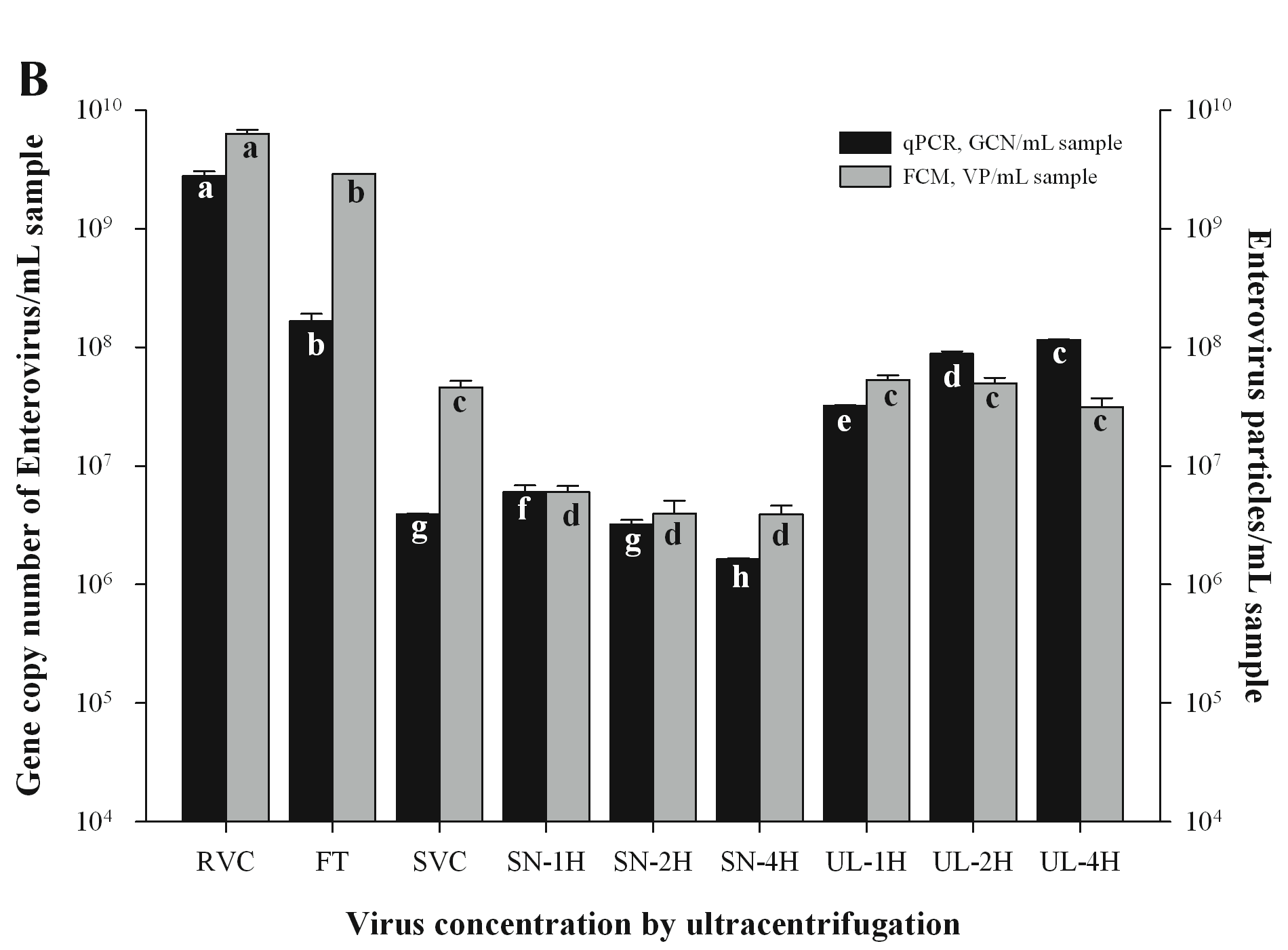


**Fig. S1** Quantitation of adenoviruses (A) and enteroviruses (B) using quantitative PCR and flow cytometry. Recovery efficiency was evaluated for both supernatant and concentrated pellets at different time points (1 h, 2h, and 4h) of the ultracentrifugation process. RVC: raw viral concentrate; FT: viral particles filtrated through a 0.2-μm membrane filter; SVC: spiked viral concentrate with MEM; SN-1H: supernatant collected after 1h of ultracentrifugation; SN-2H: supernatant collected after 2h of ultracentrifugation; SN-4H: supernatant collected after 4h of ultracentrifugation; UL-1H: ultracentrifugate collected after 1h of ultracentrifugation; UL-2H: ultracentrifugate collected after 2h of ultracentrifugation; UL-4H: ultracentrifugate collected after 4h of ultracentrifugation. Black bars represent the mean GCNs per mL of sample using qPCR approach at each time point of the ultracentrifugation process (n=3). Gray bars represent the mean of viral particles per mL of sample using flow cytometry (n=2). Error bars indicate standard deviations. Means with different letters indicate statistical significance at the 0.05 level.


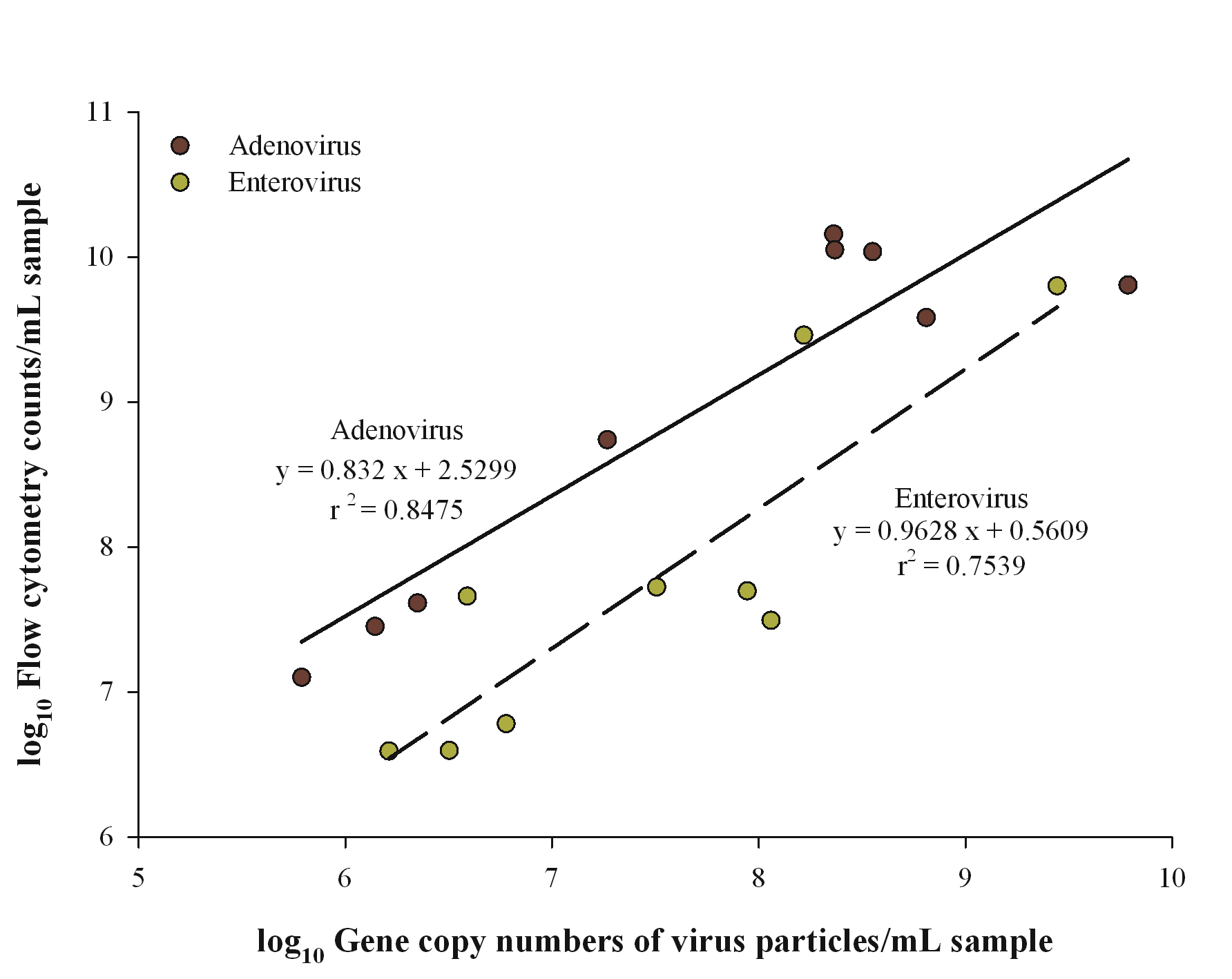


**Fig. S2** Relationship between quantitative PCR and flow cytometry approaches to quantify viral particles of adenoviruses (represented by the solid regression line and brown filled circles) and enteroviruses (represented by the dotted regression line and green filled circles).

| 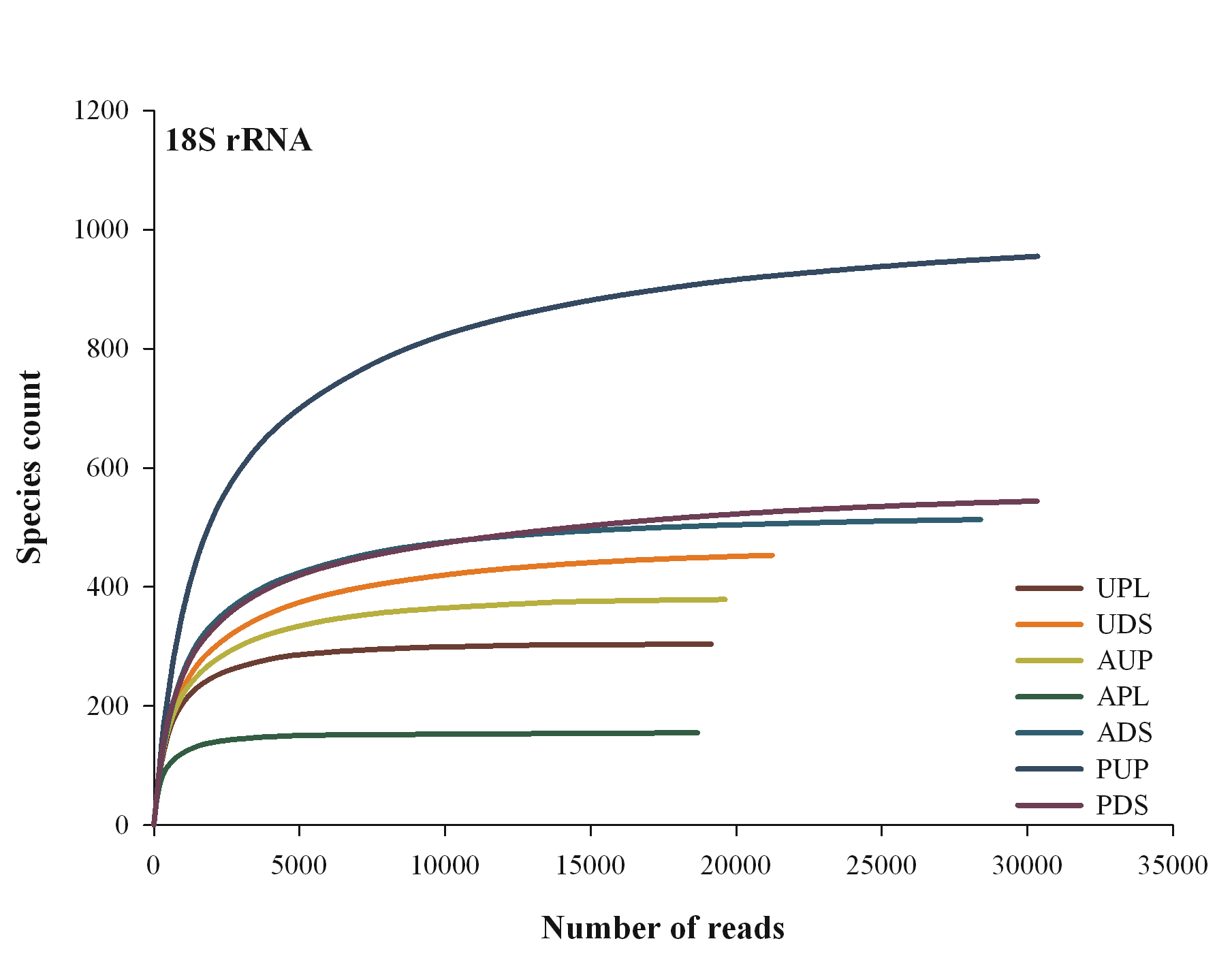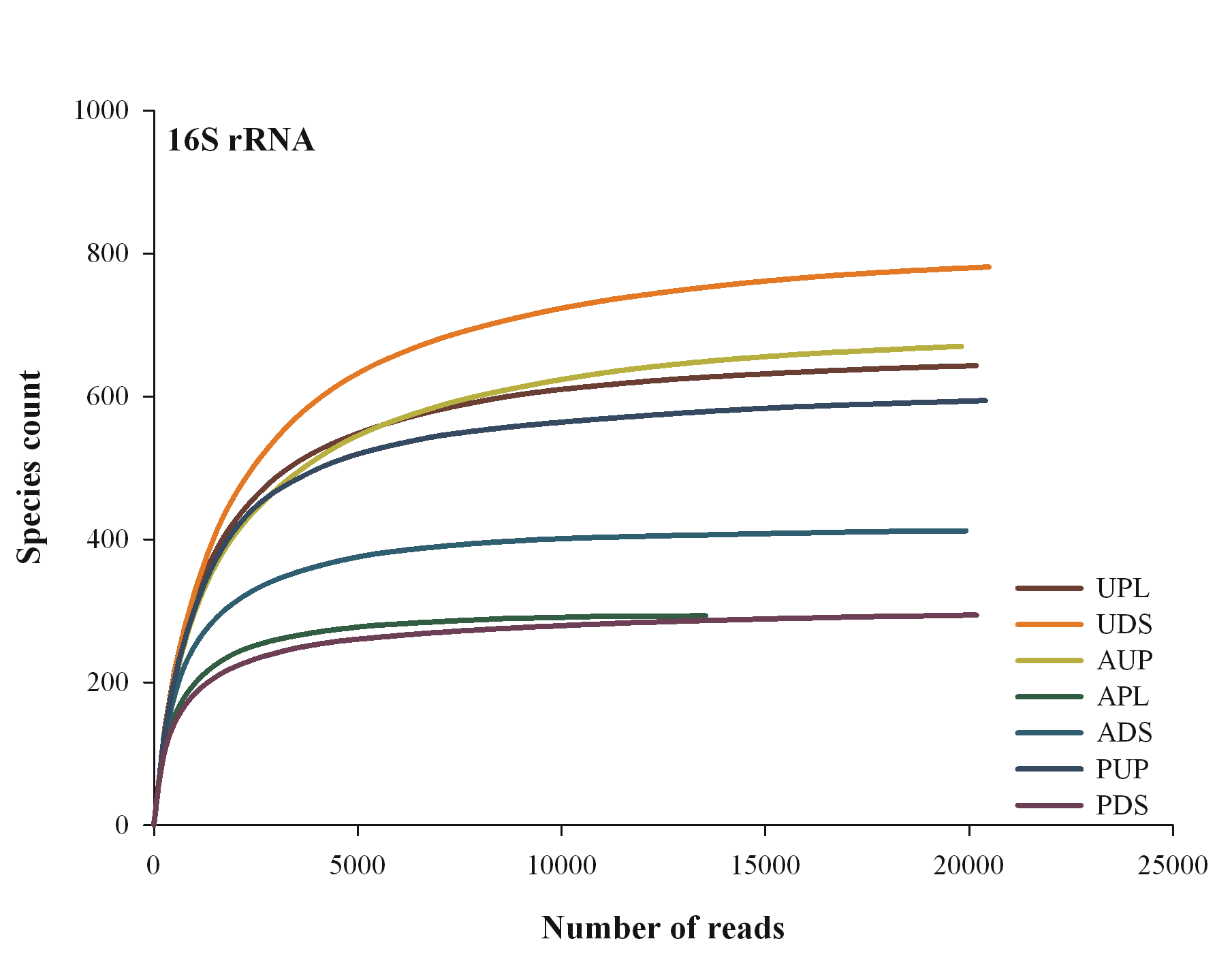 | 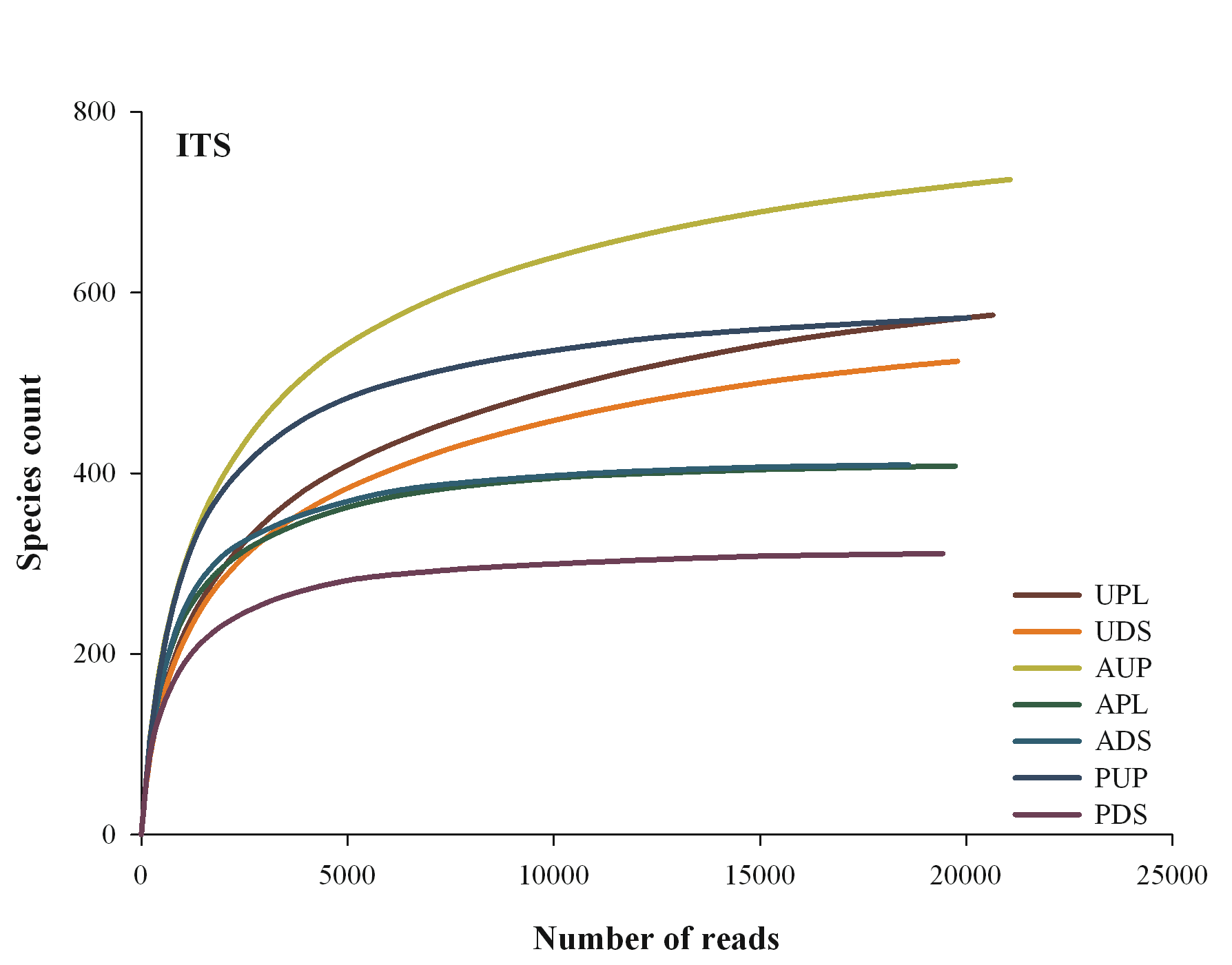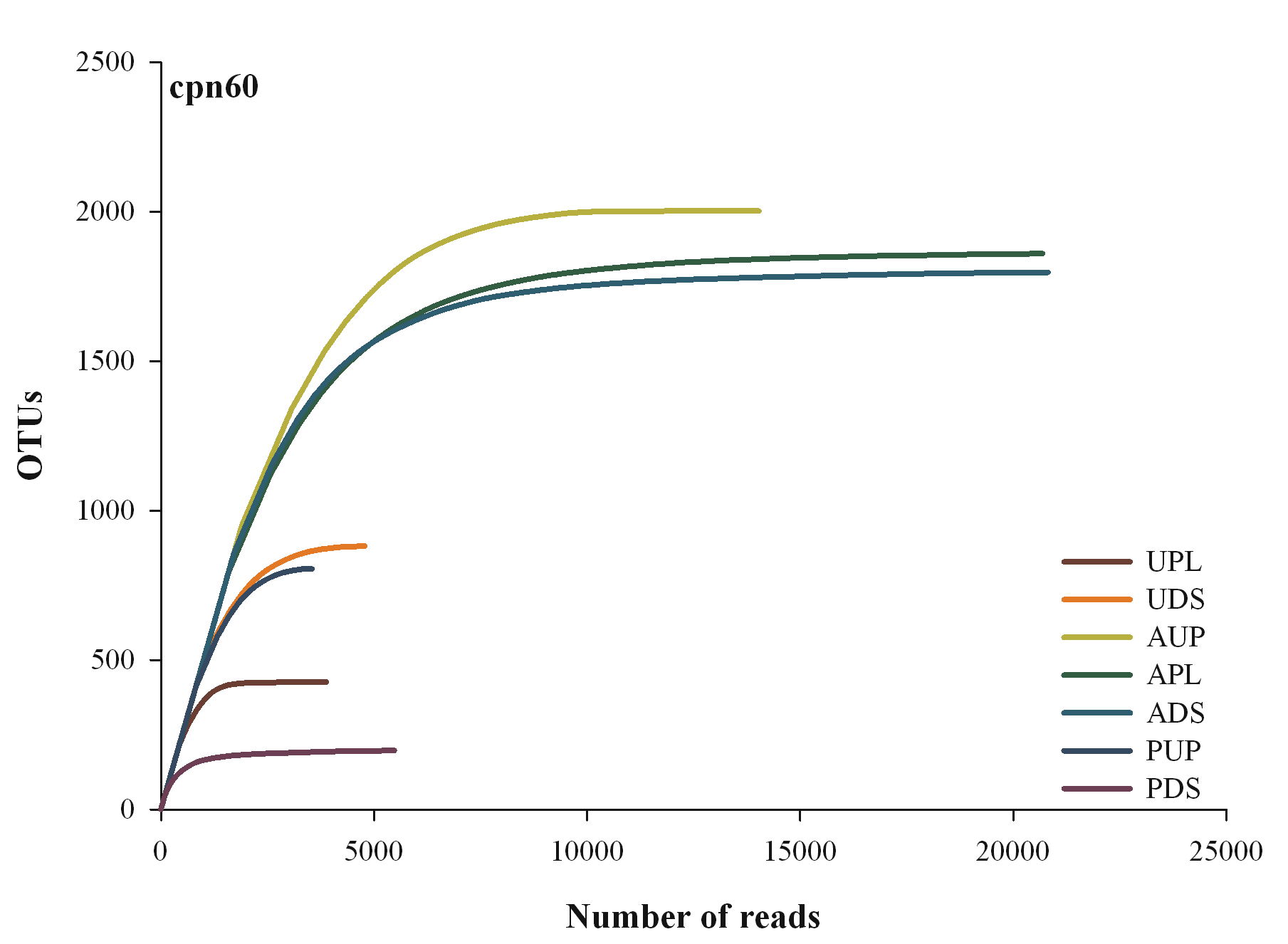 |
| --- | --- |
| 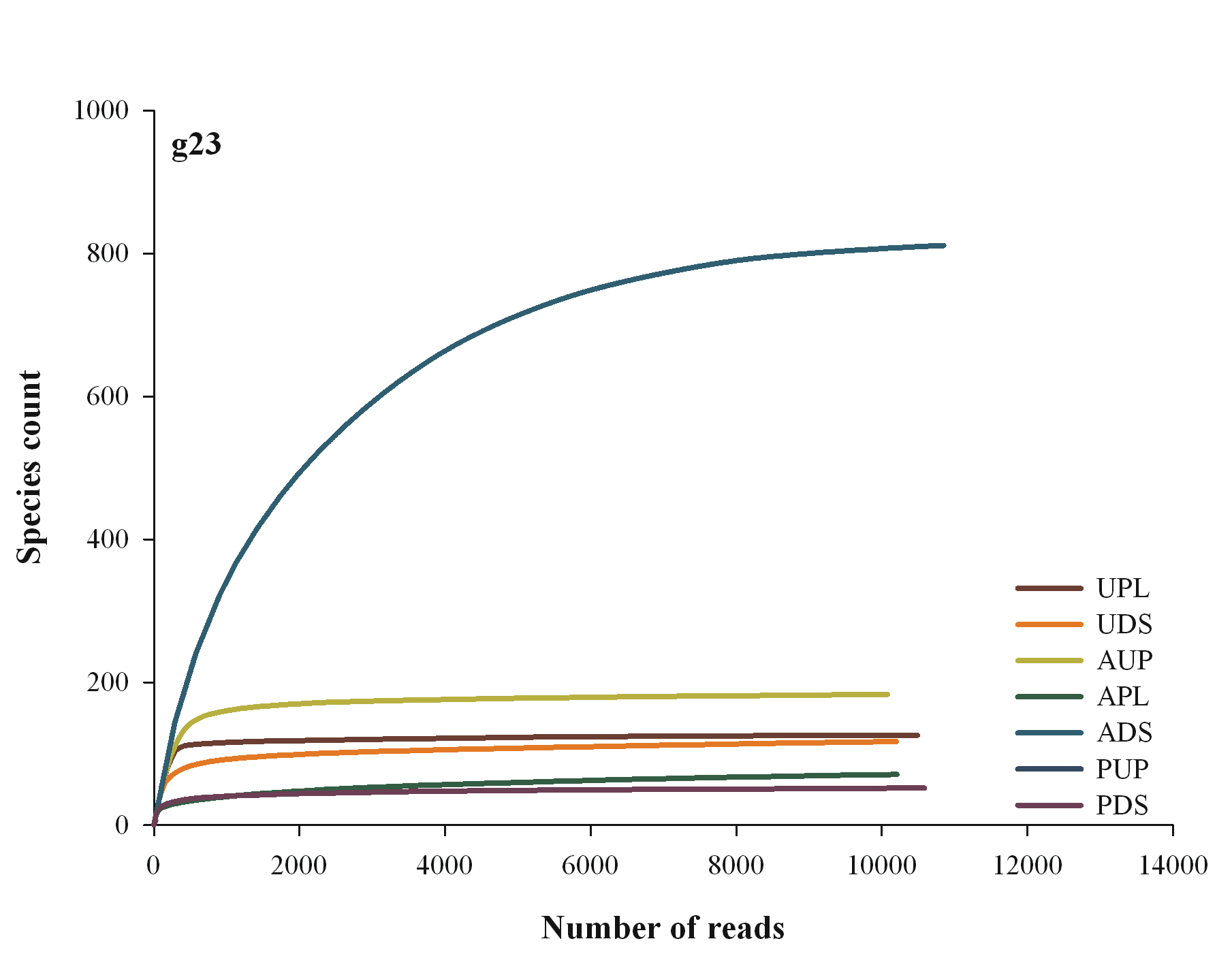 | 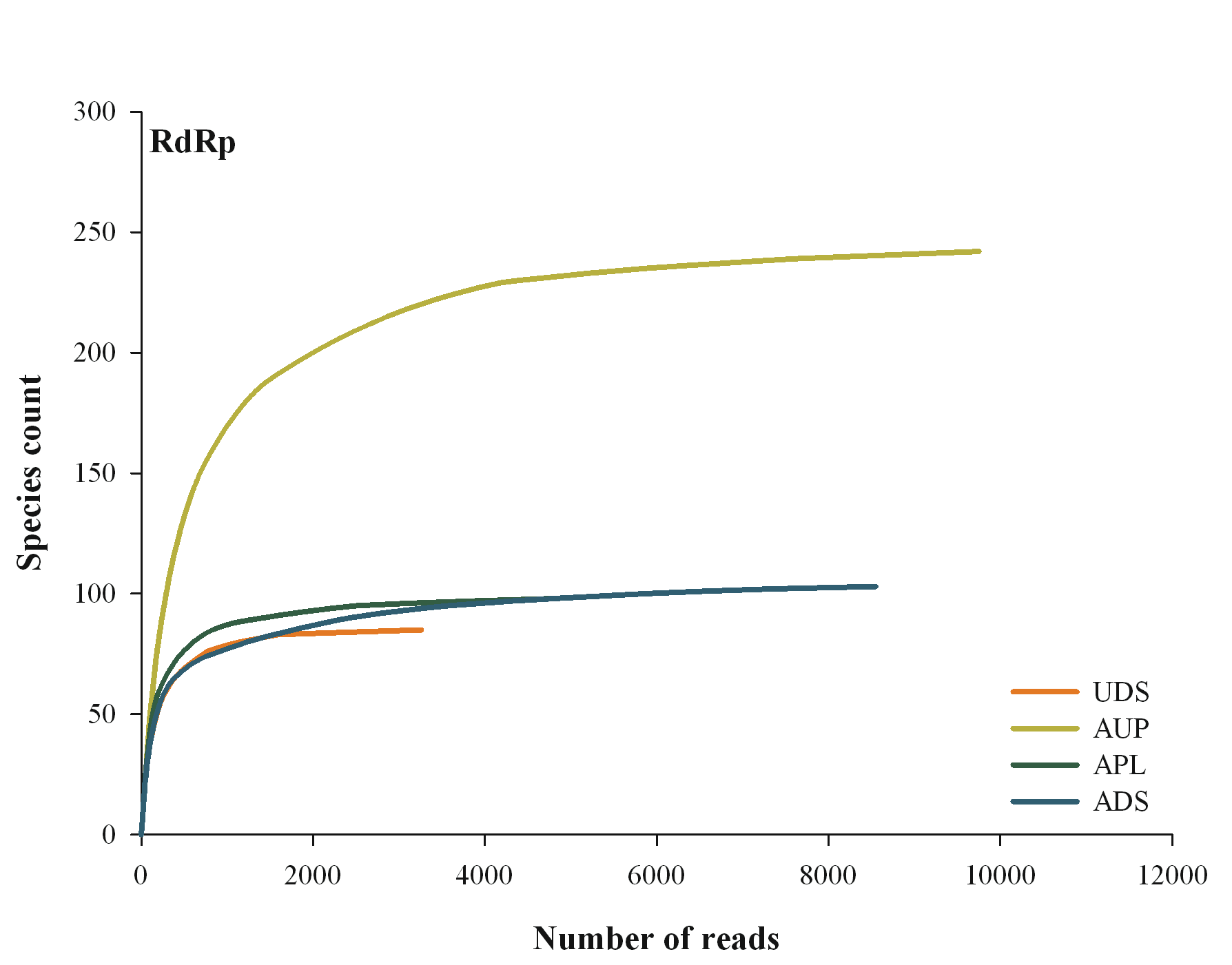 |

**Fig. S3** Rarefaction analysis of amplicon sequences: 18S rRNA, ITS, 16S rRNA, *cpn*60, *g23*, and RdRp observed in watershed sites. UPL: urban polluted; UDS: urban downstream; AUP: agricultural upstream site; APL: agricultural polluted; ADS: agricultural downstream; PUP: protected upstream; PDS: protected downstream.


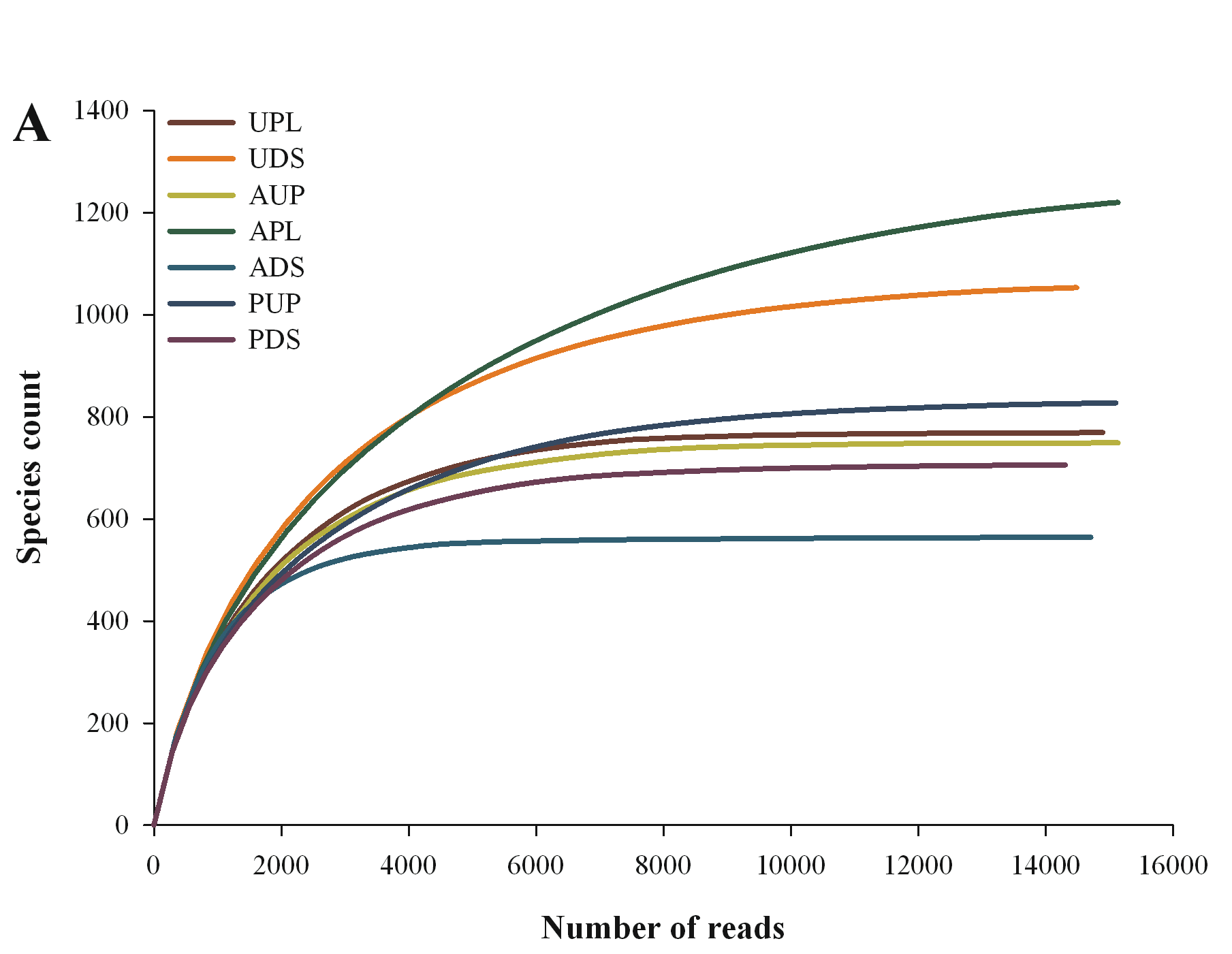


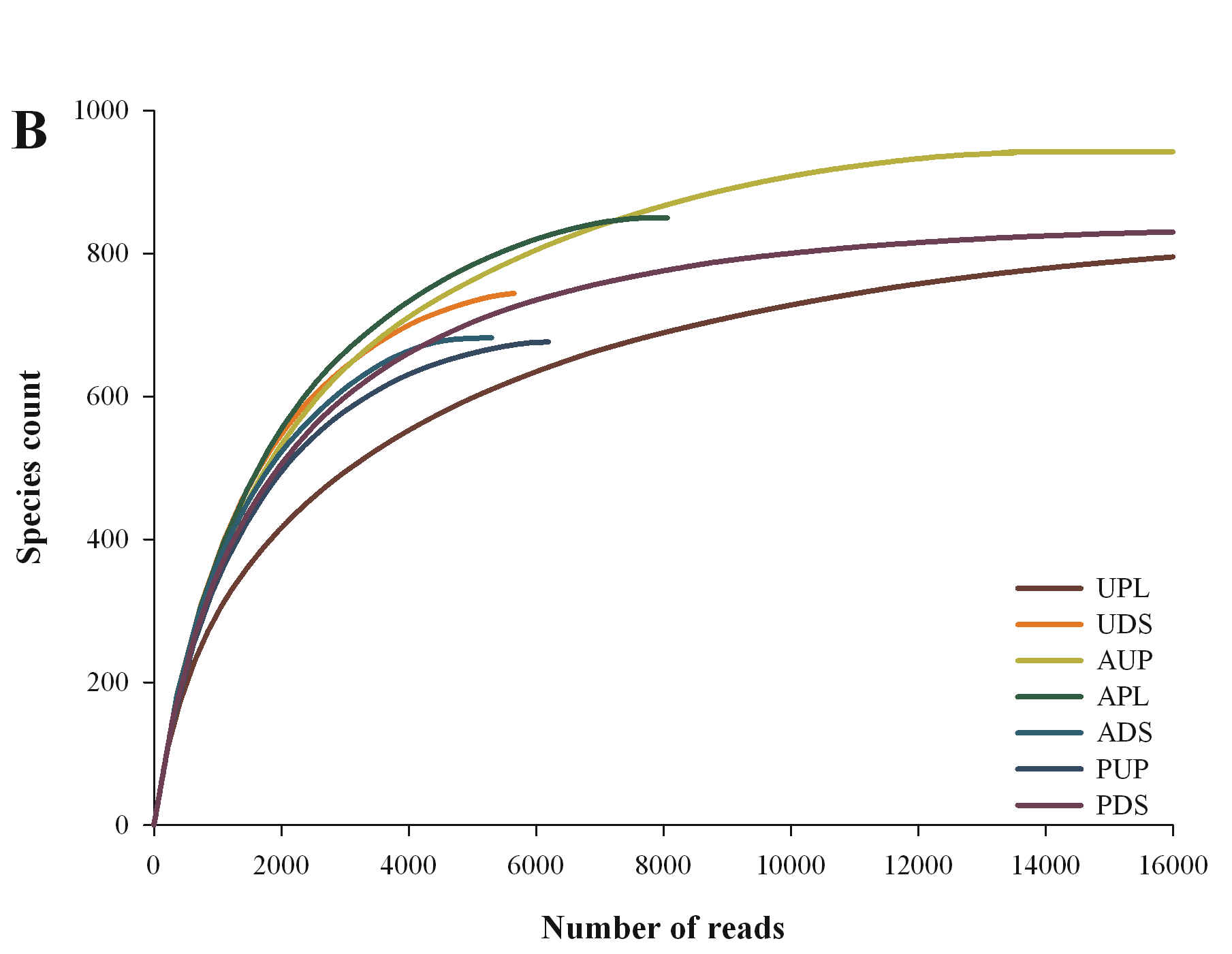


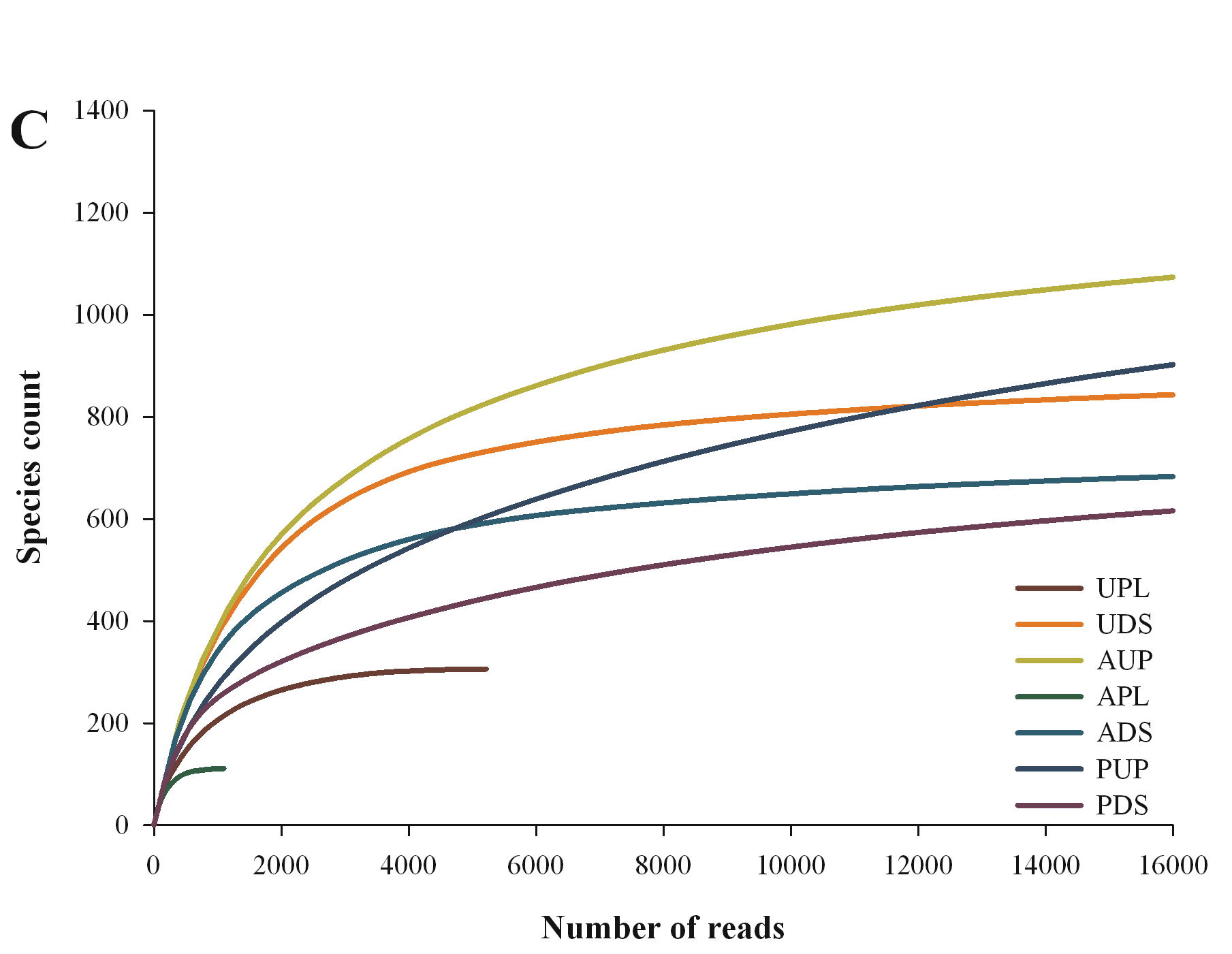


**Fig. S4** Rarefaction analysis of A) bacterial, B) viral DNA, and C) viral RNA communities observed in watershed sites. UPL: urban polluted; UDS: urban downstream; AUP: agricultural upstream site; APL: agricultural polluted; ADS: agricultural downstream; PUP: protected upstream; PDS: protected downstream.


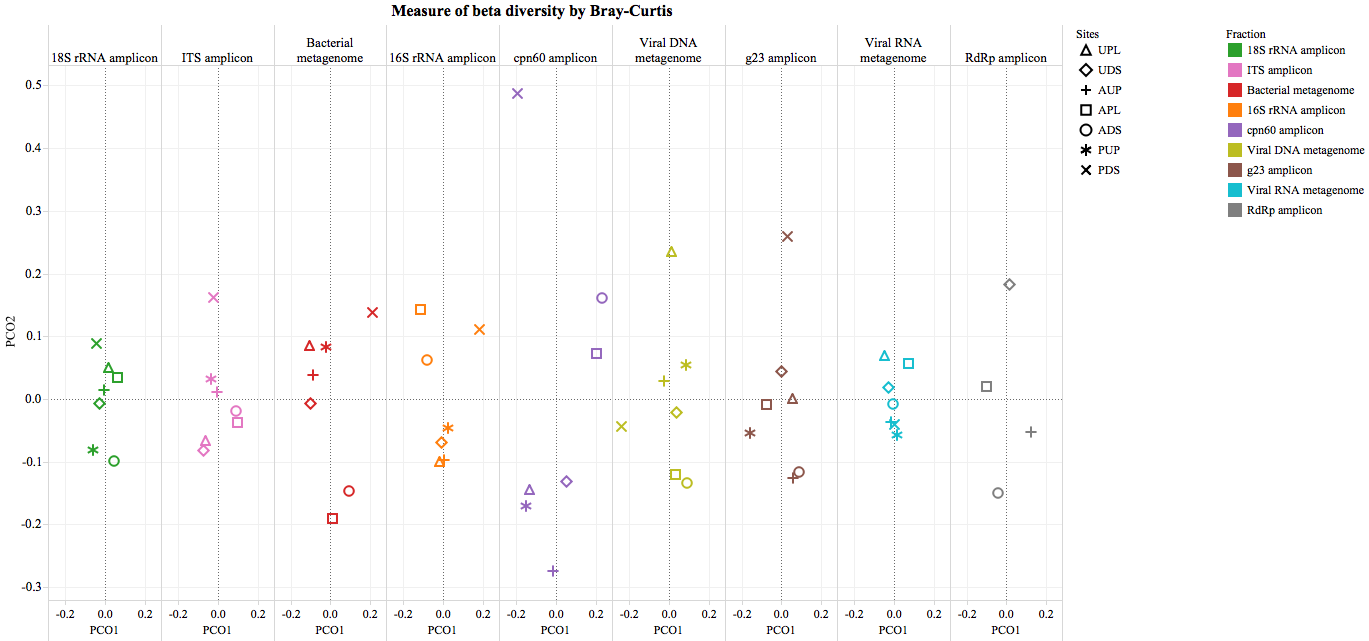


**Fig. S5** Principal coordinate analysis using Bray-Curtis dissimilarity measures (beta diversity) for metagenomes and amplicons in watershed locations. UPL: urban polluted; UDS: urban downstream; AUP: agricultural upstream site; APL: agricultural polluted; ADS: agricultural downstream; PUP: protected upstream; PDS: protected downstream.
